# Using Medication Management Technologies in Swiss Primary Care: Mixed Methods Study

**DOI:** 10.2196/68857

**Published:** 2025-08-27

**Authors:** Jeanne Maria Wildisen, Alessia Romer, Martina Zangger, Benjamin Bugnon, Sven Streit, Kristie Rebecca Weir, Katharina Tabea Jungo

**Affiliations:** 1 Institute of Primary Health Care (BIHAM), University of Berne, Berne, Switzerland; 2 Graduate School for Health Sciences, University of Berne, Berne, Switzerland; 3 Institute of Pharmaceutical Sciences of Western Switzerland, University of Geneva, Geneva, Switzerland; 4 Faculty of Medicine and Health, Sydney School of Public Health, The University of Sydney, Sydney, Australia; 5 Center for Healthcare Delivery Sciences (C4HDS), Division of Pharmacoepidemiology and Pharmacoeconomics, Department of Medicine, Brigham and Women's Hospital and Harvard Medical School, 1620 Tremont St, Boston, MA, 02120, United States, 1 857-307-3812

**Keywords:** medication management information technologies, medication optimization, Swiss Electronic Patient Record, shared electronic medication plans, primary care, older adults, primary care physicians

## Abstract

**Background:**

Medication management IT, such as shared electronic medication plans (eMediplan) and the Swiss Electronic Patient Record, is increasingly rolled out across Switzerland. They can support primary care physicians and older adults to optimize medication use and reduce medication-related harm. Understanding users’ expectations is essential for the implementation of medication management IT in primary care settings.

**Objective:**

This study aims to explore primary care physicians’ and older adults’ experiences and attitudes regarding medication management IT and identify barriers and facilitators to their use.

**Methods:**

We used a convergent mixed methods design using internet-based questionnaires and semistructured interviews with primary care physicians and older adults in Swiss primary care settings from January to August 2024. Participants included older adults aged ≥60 years who were using ≥2 prescribed medications daily, as well as primary care physicians practicing in Switzerland. Quantitative questionnaire data were analyzed using descriptive statistics to describe current use and attitudes regarding medication management IT. Qualitative data were analyzed using thematic analysis.

**Results:**

A total of 252 older adults (n=126, 50.0% female; mean age 73, SD 7 years) and 46 primary care physicians (n=15, 32.6% female; mean age 54, SD 11 years) completed the questionnaire. Notably, 7/252 (2.8%) older adults and 21/46 (45.7%) physicians reported using shared electronic medication plans. Most older adults reported not using the Swiss Electronic Patient Record (240/252, 95.2%) but expressed willingness to adopt it to manage (164/240, 68.3%) or share (179/240, 74.6%) their health information in the future. Most physicians were open to using digital tools for medication optimization (35/46, 76.1%) or a platform to coordinate medication optimization with patients and other health care providers (29/46, 63.0%). Interviews were conducted with 19 older adults (12/19, 63.2% female; mean age 77, SD 9 years) and 16 physicians (5/16, 31.3% female). The qualitative data helped explain the quantitative findings. Older adults rarely used medication management IT, while physicians mainly used basic tools integrated into their practice information system (eg, interaction checkers). Barriers and facilitators for both groups included information about these novel technologies, accessibility, perceived need and benefit, usability and accessibility, data protection, and required time and effort.

**Conclusions:**

Although older adults and primary care physicians perceived advantages of medication management IT, current use remains limited. Improving access, usability, and training for all stakeholders may facilitate broader adoption and enhance medication safety in interprofessional primary care settings.

## Introduction

An increasing number of older adults with complex health care needs regularly use multiple medications. Globally, nearly 40% of individuals aged ≥60 years are affected by polypharmacy, defined as the use of 5 or more medications [1,2]. Polypharmacy is associated with functional decline, decreased quality of life, and reduced medication adherence, leading to negative health outcomes and increased health care costs [3,4]. Over 45% of older adults with polypharmacy are using at least 1 potentially inappropriate medication, where risks (eg, adverse drug reactions and hospitalization) outweigh benefits [5,6]. With aging populations, optimizing medications and minimizing medication-related harm is increasingly crucial [7]. Given that primary care physicians (PCPs) often see their patients for many years and take a key role in coordinating their care, they play a vital role in managing and optimizing medications [8]. In this study, PCP refers to general practitioners practicing in Switzerland. Typically, they are physicians specializing in general internal medicine who provide frontline primary care.

With the digitalization of health care, medication management IT offers promising solutions to help address challenges faced by our societies, such as inappropriate polypharmacy. In this paper, medication management IT refers to electronic tools and software that support health care professionals and patients throughout the medication management process, ranging from prescribing, transmitting of prescribing information, dispensing, and administering, to medication monitoring [9,10]. Examples of such technologies include drug interaction checkers, clinical decision support systems, shared electronic medication plans, electronic patient records, electronic pill reminders, and dosing aids. Medication management IT can improve prescribing and monitoring processes, reduce medication errors, and increase adherence among older adults [9,11-13]. Electronic exchange of medication information fosters interprofessional collaboration and prevents communication problems, like contradictory information from different health care providers or discrepancies in medication lists [14-18]. A growing number of technologies available for self-monitoring and self-care can empower patients to take a more active role in their health [19].

The design, implementation, and adoption of medication management IT vary widely across and within countries. While countries like the United Kingdom and the Netherlands have made significant strides in digitalizing primary care, others, namely, Switzerland, are slower [20,21]. Nevertheless, Swiss primary care settings are increasingly digitalized. While 35% of PCPs kept their patients’ medical records electronic in 2013, the percentage had risen to 72% in 2022 [22,23]. Electronic medical records are used by individual health care providers to document and manage patient information within a single practice or health care organization [24]. In recent years, new digital technologies to support medication management and optimization have been introduced in Switzerland. Examples include shared electronic medication plans (called eMediplan) and the Swiss Electronic Patient Record, which are interoperable tools designed to facilitate the exchange of medication and health information among health care providers and patients [25,26]. In the Swiss context, Electronic Patient Records are a national digital health system that allows patients to access, share, and control access to their data (German: elektronisches Patientendossier; French: dossier électronique du patient). They are currently voluntary and initiated by patients, who determine which health care providers can access their health information [26]. Other countries are implementing similar solutions for both shared electronic medication plans and electronic patient records with varying architectures, functionalities, information sources, and degrees of voluntariness of participation [27-30]. Their success depends on organizational, human, and technological factors within a specific health care setting [31].

To address gaps in using medication management IT in Swiss primary care settings, this mixed methods study using quantitative surveys and qualitative semistructured interviews aimed to explore the experiences and attitudes of PCPs and older adults towards these technologies. Better understanding barriers and facilitators will help inform the future implementation of medication management IT in the Swiss context. In both the surveys and interviews, the eMediplan and the Swiss Electronic Patient Record were used as illustrative examples of novel medication management IT rolled out across Swiss primary care settings.

## Methods

### Study Design

This study used a convergent mixed methods design, in which quantitative and qualitative data were collected in Swiss primary care settings [32]. Such a mixed methods approach has previously been used by others to study the adoption of new technologies [33-36]. We reported this study following the COREQ (Consolidated Criteria for Reporting Qualitative Research) (Checklist 1) and the CHERRIES (Checklist for Reporting Results of Internet E-Surveys) checklist [37,38] (Checklist 2).

### Data Collection

We collected quantitative cross-sectional data through online-administered questionnaires for older adults and PCPs in Swiss primary care settings and qualitative data through semistructured interviews with both groups. Older adults had to be between 60 and 100 years of age, and physicians had to practice in Swiss primary care settings.

#### Quantitative Internet-based Questionnaires

The questionnaire for older adults, available in German and French, contained 23 multiple-choice, open, or Likert scale questions (Section A in Multimedia Appendix 1). The German version was created by the study team and subsequently translated into French, followed by back-translation into German to verify accuracy. The item generation was done by the study team and guided by the study objectives. One question regarding the patient’s role in medication decisions was adapted from the Control Preferences Scale [39]. Two eligible older adults piloted the questionnaire. Older adults were recruited through the YouGov Switzerland (formerly LINK) online research panel, which currently comprises over 115,000 active panelists. Panel members were initially recruited via population-representative telephone surveys using randomly generated mobile phone numbers (and until 2019, also registered landline numbers), resulting in a theoretical coverage of approximately 97%. Only individuals without personal telephone access (eg, residents of correctional facilities) were excluded. Panel members are thoroughly profiled during the recruitment process and subsequently invited to participate in surveys via email based on relevant study criteria [40]. Each question had to be answered for the respondent to submit the questionnaire, and respondents could return to previous questions. The PCP questionnaire was developed by the study team and had 2 parts (one on digital tools to support medication optimization, used for this paper, and one on a digital platform to optimize hypertension treatment). Two questions of the questionnaire used in this study were based on elements from existing literature, the remaining questions were developed specifically for this study [41]. Participants were randomly assigned to one of the 2 parts of the questionnaire (participants were asked whether their date of birth was on an even or odd day). It was administered through Survey Monkey, available in German, French, and Italian and contained 46 multiple-choice, open, or Likert scale questions (Section B in Multimedia Appendix 1) [42]. The German questionnaire was translated into French and Italian and then independently back-translated to verify the translation accuracy. Five eligible PCPs piloted the questionnaire. The link to the internet-based questionnaire was distributed through newsletters of professional clinical societies (Swiss Society of General Internal Medicine (SSGIM), Swiss Young Family Doctors (Junge Haus- und KinderärztInnen Schweiz), EQUAM Foundation), advertising on the web platform Swiss Health Web, and snowball sampling. The questionnaire was accessible with the corresponding link. The survey contained both mandatory and optional questions, and respondents could return to previous questions. Some questions were optional based on previous answers. Each IP address was allowed to complete the questionnaire once. Data were collected between January and August 2024.

#### Qualitative Semistructured Interviews

A female medical doctoral student (JMW) conducted one-to-one semistructured interviews in German with older adults and PCPs in person (researchers’ workplace, older adults’ homes, and primary care practices) or remotely via Zoom (Zoom Video Communications) [43]. We recruited older adults through convenience sampling by distributing flyers in nursing homes, assisted living facilities, through home care services and primary care practices, personal contacts, and snowball sampling. As a result, mainly older adults from the German-speaking Bern and Lucerne regions were recruited. We recruited physicians using convenience sampling through the network of study team members, phone calls and emails to primary care practices, and snowball sampling. Accordingly, half of the PCPs were recruited from the Bern region, and the other half from across the rest of German-speaking Switzerland. Interview guides with open and probing questions were developed by the study team and piloted with 2 eligible older adults and physicians each (Section C and D in Multimedia Appendix 1). In addition, a “cheat sheet,” which defined important terms (eg, eMediplan) was provided to the interviewees to clarify definitions (Section E in Multimedia Appendix 1). At the beginning of each interview, participants were informed of the study’s purpose and that the results would contribute to JMW’s medical doctoral thesis. Interviews were audio-recorded with participant consent. The interviews took place between February and July 2024, until data saturation was reached. The median interview duration was 49 (IQR 29‐114) minutes for patients and 46 (IQR 31‐62) minutes for physicians.

### Data Analysis

#### Quantitative Data

We used descriptive statistics to describe older adults’ and PCPs’ characteristics and main outcomes. Data were described as counts (n) and percentages (%) and means and SDs. We used RStudio version 2023.06.2+561 (Posit, PBC) for the analysis [44]. We reported the missingness of the variables.

#### Qualitative Data

Audio recordings of the interviews were transferred to MAXQDA software version 24.2.0 (VERBI Software GmbH) [45] for transcription and coding. The recordings were transcribed manually and verbatim to ensure accuracy and data integrity. Thematic analysis was conducted separately for the transcripts of older adults and PCPs. After data familiarization, a separate codebook for each group was created with codes that capture important aspects of the data for the research questions (Tables S1 and S2 in Multimedia Appendix 1) and the codebooks were refined as necessary throughout the process [46]. To enhance reflexivity and consistency, coding decisions were made through an iterative, shared process between researchers (JMW and KTJ), with discrepancies discussed and resolved collaboratively. MAXQDA’s functionality to view coded segments within their original transcript context enabled a more thorough and contextualized interpretation, thereby enhancing the trustworthiness of the findings. Themes were identified based on patterns across the coded data and were reviewed, refined, and named in further steps. Representative quotes were selected to illustrate key findings.

#### Data Triangulation

After analyzing the quantitative and qualitative data separately, we used qualitative data to explain and provide context for the quantitative findings (Table S3 in Multimedia Appendix 1).

### Ethical Considerations

#### Ethical Approval

The competent ethics committee (Ethics Committee of the Canton of Bern) declared the project not to fall under the Human Research Act [47] and therefore waived its approval (Req-2023‐00302).

#### Informed Consent

All interviewees gave their written informed consent to be interviewed and for the interview to be audio recorded. The study information letter outlined the study’s purpose, eligibility criteria, procedures, time commitment, potential benefits and risks, participant rights and obligations, data confidentiality, withdrawal options, compensation, and funding. Survey participants gave their informed consent by clicking on the “Continue” button after the first survey page with information about the content and duration, anonymity and confidentiality, and purpose of the survey.

#### Privacy and Confidentiality Protection

Survey data were collected anonymously. At the end of the PCP survey, PCPs could voluntarily leave an email address to take part in the prize draw for a CHF 50 voucher (US$ 62 as of July 2025). Audio recordings and interview transcripts were encrypted, and the decryption was stored securely on a protected local database only JMW and KTJ could access. Transcripts or findings were not returned to participants for comment.

#### Compensation

Interview participants received a CHF 50 supermarket voucher (US$ 62 as of July 2025). Two CHF 50 vouchers were randomly drawn among the participants in the PCP survey who voluntarily provided their contact information.

### Research Team

Our research team brings diverse expertise and perspectives that shaped our approach to this study. KRW and KTJ have extensive experience in qualitative research, which informed our methodological choices and interpretation of participants’ perspectives. KTJ, KRW, and SS specialize in medication optimization. KTJ, SS, BB, and JMW specialize in digital tools for medication optimization. JMW, SS, MZ, AR, and BB have clinical practice experience, ensuring that our findings remain relevant and applicable to real-world health care settings. Our collective values emphasize patient-centered care, innovation, and evidence-based decision-making, which influenced how we designed, conducted, and interpreted our research.

## Results

### Quantitative Online Questionnaires

#### Participants

Out of 322 older adults who started the questionnaire, 252 completed the survey (Figure S1 in Multimedia Appendix 1). Among them, 126/252 (50.0%) were female, their average age was 73 (SD 7) years, and they used an average of 4 (SD 2) prescription medications per day. The majority (158/252, 62.6%) did not wish for more information about the risks and benefits of medications. Most patients (235/252, 93.3%) were (very) satisfied with their role when discussing medication changes with their PCP (Table 1). Out of 121 PCPs who started the questionnaire, 5 did not meet the inclusion criteria. A total of 46 PCPs completed the survey part relevant to this project (Figure S2 in Multimedia Appendix 1). Overall, 15/46 (32.6%) were female, with an average age of 54 (SD 11) years, and they had an average of 19 (SD 12) years of professional experience as PCPs. Notably, 39/46 (84.8%) of them were German-speaking, 20/46 (43.5%) had their practice in an urban area, and most of them (41/46, 89.1%) rated their own skills in using digital devices as (very) good. Only 1/46 (2.2%) participant kept patient records exclusively on paper (Table 2). Age and work experience were not perfectly normally distributed, but means and medians were similar across groups (eg, PCPs’ age: mean 54.5, SD 11 and median 55.0, IQR 47-60; PCPs’ work experience: mean 18.6, SD 12 and median 20.0, IQR 7.5-26.5; older adults’ age: mean 73.5, SD 7 and median 73.0, IQR 68-78).

**Table 1.** Baseline characteristics of older adults (n=252).^a^

Characteristics	Value
Age (years), mean (SD)	73 (7)
Gender, n (%)	
Female	126 (50)
Number of prescription medications, mean (SD)	4 (2)
Wish for more information about the risks and benefits of medications, n (%)	
Strongly disagree	38 (15.1)
Disagree	120 (47.6)
Agree	58 (23.0)
Strongly agree	30 (11.9)
I do not know	6 (2.4)
Satisfaction with your own role in discussions with your primary care physician about changes to your medication list, n (%)	
Very unsatisfied	1 (0.4)
Unsatisfied	6 (2.4)
Neutral	10 (4.0)
Satisfied	108 (42.9)
Very satisfied	127 (50.4)

a No missing data.

**Table 2.** Baseline characteristics of primary care physicians (n=46).

Characteristics	Value
Age (years)^a^, mean (SD)	54 (11)
Gender^b^, n (%)	
Female	15 (33.3)
Work experience as a primary care physician in years^a^, mean (SD)	19 (12)
Language spoken in the practice^b^, n (%)	
German	39 (84.8)
French	5 (10.9)
Italian	2 (4.3)
Location of the practice^b^, n (%)	
Urban area	20 (43.5)
Suburban area	13 (28.3)
Rural area	13 (28.3)
How would you rate your skills in using digital devices (eg, computer and smartphone)?^b^ n (%)	
Very poor	0 (0)
Poor	2 (4.3)
Neither good nor poor	3 (6.5)
Good	22 (47.8)
Very good	19 (41.3)
Use of electronic medical records in practice^b^, n (%)	
Patients’ medical records are completely electronic	42 (91.3)
Patients’ medical records are partly electronic	3 (6.5)
Patients’ medical records are kept exclusively on paper	1 (2.2)

a <7.0% missing data.

b No missing data.

#### Medication Management

In total, 146/252 (57.9%) older adults had a medication plan, but only 7/252 (2.8%) had a shared electronic medication plan. Of those with a shared electronic medication plan, 133/146 (91.1%) were (very) satisfied with it while 70/106 (66.0%) of those without a shared electronic medication plan did not want one from their PCP. Only 12/252 (4.8%) actively used the Swiss Electronic Patient Record (Table 3). Overall, 21/46 (45.7%) PCPs prepared shared electronic medication plans. Of the 24 reporting not to prepare shared electronic medication plans, 7/24 (29.2%) stated they could not for technical reasons, and 10/24 (41.7%) for other reasons. Notably, 9/46 (19.6%) had access to the Swiss Electronic Patient Record (Table 4). For most PCPs, the practice information system enabled the creation of medication lists (43/46, 93.5%), medication plans (41/46, 89.1%), and the issuing of electronic prescriptions (32/46, 69.6%). Furthermore, 35/46 (76.1%) reported using electronic and nonelectronic tools supporting medication optimization (eg, drug interaction checker and guidelines) (Table 5).

**Table 3.** Older adults’ use of medication plans, shared electronic medication plans^a^ , and the Swiss Electronic Patient Record^b^ (n=252)^c^.

Survey items	Value
Availability of a plan with all your current medications, n (%)	
I do not have a medication plan	106 (42.1)
I make my own medication plan	76 (30.2)
I receive a medication plan from my primary care physician	60 (23.8)
I receive a shared electronic medication plan from my primary care physician	7 (2.8)
I receive a medication plan from my pharmacy	3 (1.2)
Satisfaction with my medication plan (for those reporting to have one; n=146), n (%)	
Very dissatisfied	0 (0)
Dissatisfied	0 (0)
Neutral	13 (8.9)
Satisfied	66 (45.2)
Very satisfied	67 (45.9)
Responsibility for updating my medication plan (for those reporting to have one; n=146), n (%)	
Me or a relative takes care of updating it	74 (50.7)
My primary care physician takes care of updating it	47 (32.2)
No need to update it, I know my current medication off by heart	23 (15.8)
My pharmacy takes care of updating it	2 (1.4)
Willingness to receive a medication plan from my primary care physician (for those reporting not to have one; n=106), n (%)	
Yes	15 (14.2)
No	70 (66.0)
Undecided	21 (19.8)
Use of a Swiss Electronic Patient Record ^b^ (n=252), n (%)	
No, but I am planning to open one in the future	124 (49.2)
No, I have not opened an electronic patient record and am not planning to do so	68 (27.0)
No, I did not know that electronic patient records existed	32 (12.9)
Yes, I have one, but I do not actively use it	16 (6.3)
Yes, I use a Swiss Electronic Patient Record	12 (4.8)
Willingness to use a Swiss Electronic Patient Record to manage my own health information (for those reporting not to currently use one; n=240), n (%)	
Strongly disagree	17 (7.1)
Disagree	40 (16.7)
Agree	103 (42.9)
Strongly agree	61 (25.4)
I do not know	19 (7.9)
Willingness to use a Swiss Electronic Patient Record to share my health information with health care providers (for those reporting not to currently use one; n=240), n (%)	
Strongly disagree	11 (4.6)
Disagree	33 (13.8)
Agree	102 (42.5)
Strongly agree	77 (32.1)
I do not know	17 (7.1)

a Shared electronic medication plans are digital tools designed to manage and share medication plans electronically between health care providers and patients (eg, eMediplan in the Swiss context).

b Swiss Electronic Patient Records are a digital system that securely stores a patients’ health information, enabling authorized health care providers to access and update medical data including medication information across practice information systems.

c No missing data.

**Table 4.** Primary care physicians’ use of medication plans, shared electronic medication plans^a^ and Swiss Electronic Patient Records^b^ (n=46).

Survey items	Value
Frequency of preparing a medication plan for your patients with polypharmacy^c^, n (%)	
Never	0 (0)
Rarely	2 (4.3)
Sometimes	5 (10.9)
Often	11 (23.9)
Very often	27 (58.7)
Capability of preparing shared electronic medication plans (eMediplan) in your practice^c^, n (%)	
Yes	21 (45.7)
No	24 (52.3)
Reasons for not preparing shared electronic medication plans (eMediplan) in your practice (For those reporting not to prepare any; n=24)^d^, n (%)	
I cannot prepare shared electronic medication plans for technical reasons	7 (29.2)
I do not prepare shared electronic medication plans yet but would like to do so in the future	4 (16.7)
I do not want to prepare shared electronic medication plans	3 (12.5)
Other reasons	10 (41.7)
Practice connected to Swiss Electronic Patient Records (n=46)^c^, n (%)	
Yes	9 (19.6)
No	36 (78.3)

a Shared electronic medication plans are digital tools designed to manage and share medication plans electronically between health care providers and patients (eg, eMediplan in the Swiss context).

b Swiss Electronic Patient Records are a digital system that securely stores a patients’ health information, enabling authorized health care providers to access and update medical data including medication information across practice information systems.

c <3.0% missing data.

d No missing data.

**Table 5.** Primary care physician-reported approaches to medication optimization and use of digital tools for medication optimization (n=46).

Survey items	Value
Frequency of medication reviews for patients with polypharmacy^a^, n (%)	
Frequency far too low	1 (2.2)
Frequency too low	11 (23.9)
The frequency is just right	30 (65.2)
Frequency too high	3 (6.5)
Frequency far too high	0 (0)
Medication management tools available in the practice information system (multiple answers possible), n (%)	
Creation of medication lists (eg, simple list of patients’ current or past medications)	43 (93.5)
Creation of medication plans (eg, detailed information on medication intake for patients)	41 (89.1)
Issuing electronic prescriptions	32 (69.6)
Other tools	5 (10.9)
Use of tool(s) to support medication optimization^a^, n (%)	
Yes	35 (76.1)
No	10 (21.7)
Type of tools used to support medication reviews (multiple answers possible), n (%)	
Guidelines	28 (60.9)
Interaction checker integrated into practice information system	27 (58.7)
Online evidence-based clinical resources (eg, UpToDate)	18 (39.1)
Interaction checker outside practice information system	13 (28.3)
Lists for assessing potentially inappropriate medications (eg, Beers criteria)	11 (23.9)
Apps	8 (17.4)
Tools that can reconcile various medication lists	7 (15.2)
Electronic decision support tools	5 (10.9)
Structured templates for conducting medication reviews (digital)	4 (8.7)
Structured templates for conducting medication reviews (paper-based)	3 (6.5)
Other tools	5 (10.9)
Availability of digital tools for medication optimization (other than interaction checkers) integrated into my practice information system^b^, n (%)	
Yes	18 (39.1)
No	25 (54.3)
Willingness to use digital tools for medication optimization integrated into my practice information system^b^, n (%)	
Strongly disagree	1 (2.2)
Disagree	2 (4.3)
Neither agree nor disagree	3 (6.5)
Agree	15 (32.6)
Strongly agree	20 (43.5)
Willingness to use a digital platform to coordinate medication optimization with my patients and other health care providers^b^, n (%)	
Strongly disagree	2 (4.3)
Disagree	3 (6.5)
Neither agree nor disagree	7 (15.2)
Agree	10 (21.7)
Strongly agree	19 (41.3)

a <3.0% missing data.

b <11.0% missing data.

#### Attitude Toward Medication Management IT

**Figure 1.**
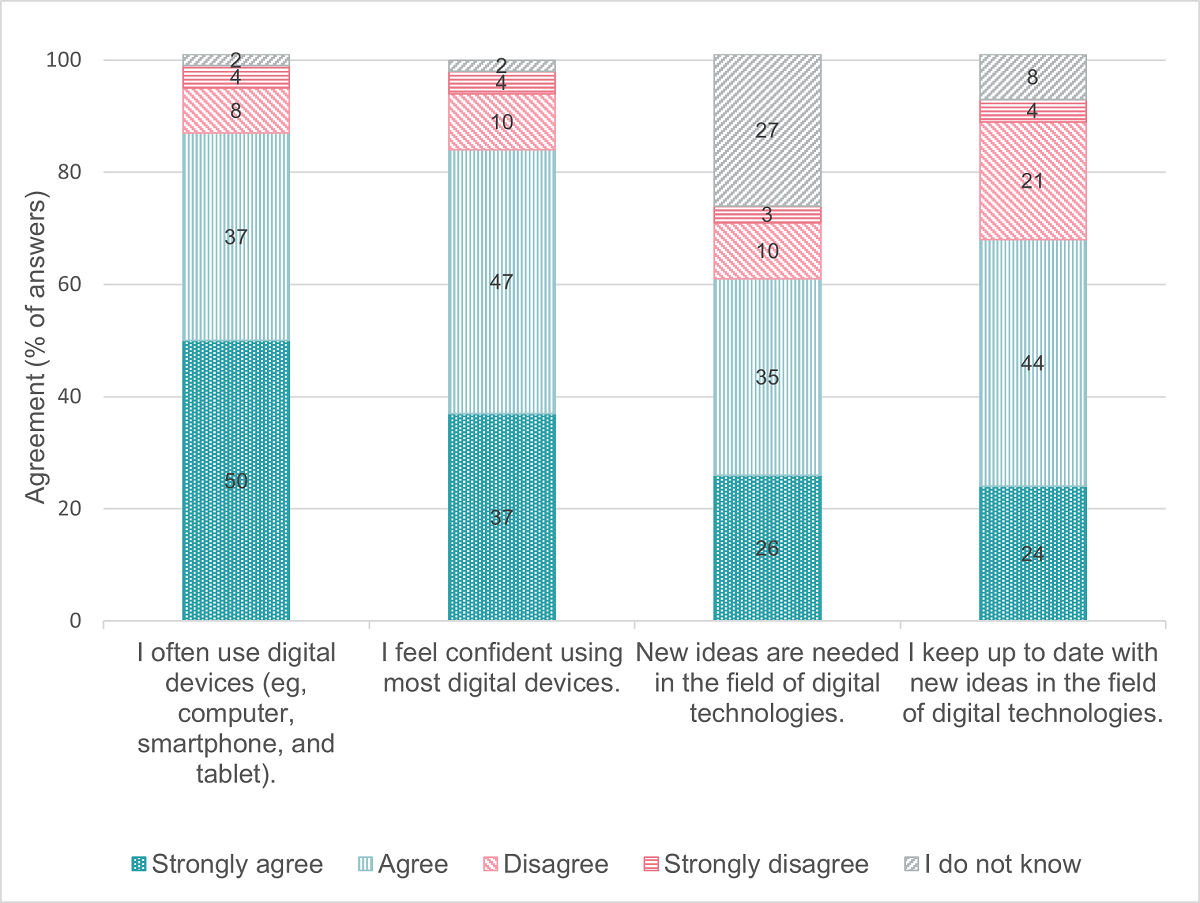
Older adults’ attitudes toward digital technologies (n=252). No missing data.

Figures1  2 indicate older adults’ mostly positive attitude toward digital technologies in daily life and toward the Swiss Electronic Patient Record. Most of the 240 older adults who reported they were not currently using a Swiss Electronic Patient Record (strongly) agreed that they would like to use one to manage health information (164/240, 68.3%) or share health information with health care providers (179/240, 74.6%) (Table 3). Most PCPs (strongly) agreed that they would like to use a digital tool for medication optimization in their practice information system (software to manage administrative, clinical, and financial operations; 35/46, 76.1%) and a digital platform to coordinate medication optimization with their patients and other health care providers (29/46, 63.0%) (Table 5). Figures3  4 show that the physicians rated multiple functions in a medication plan management tool (eg, ability to track medication plan changes) and medication optimization tool (eg, ability to monitor medication side effects) as important.

**Figure 2.**
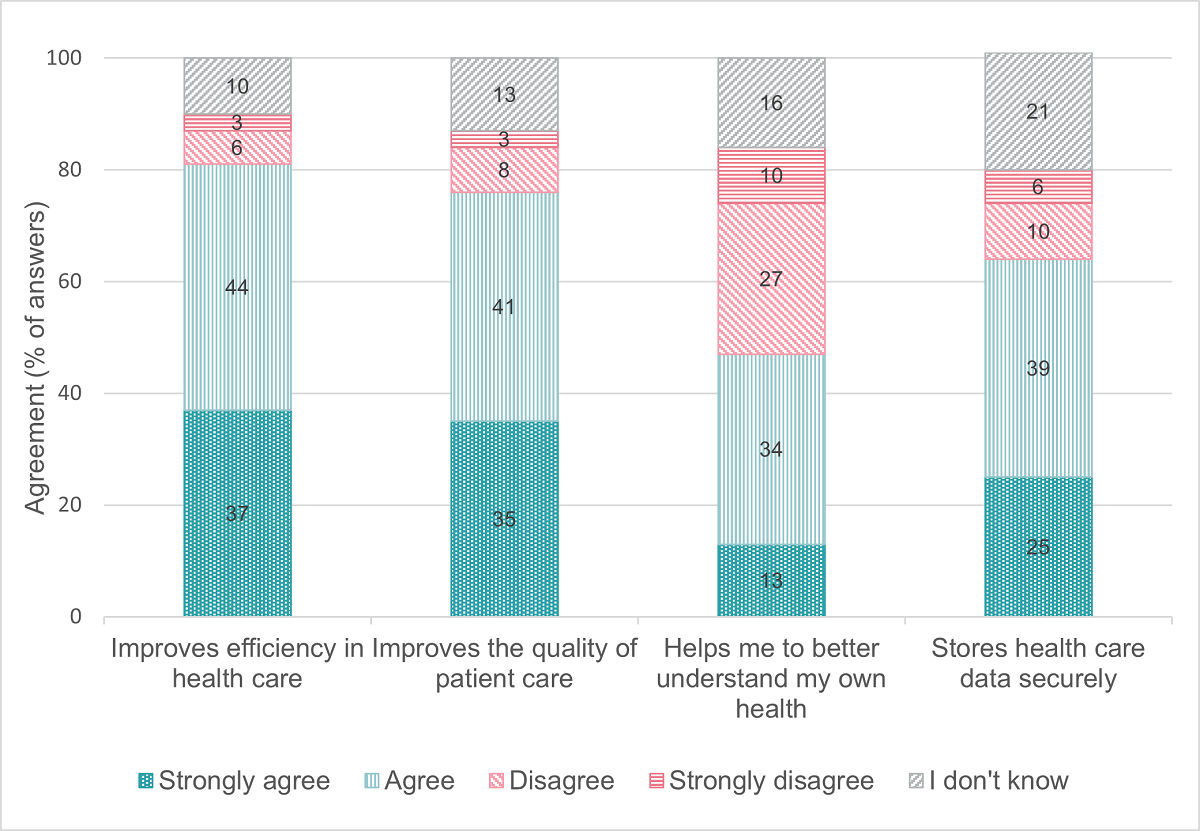
Older adults’ attitudes toward the Swiss Electronic Patient Record (n=252). Swiss Electronic Patient Records are a digital system that securely stores a patients’ health information, enabling authorized health care providers to access and update medical data, including medication information across practice information systems. No missing data.

**Figure 3.**
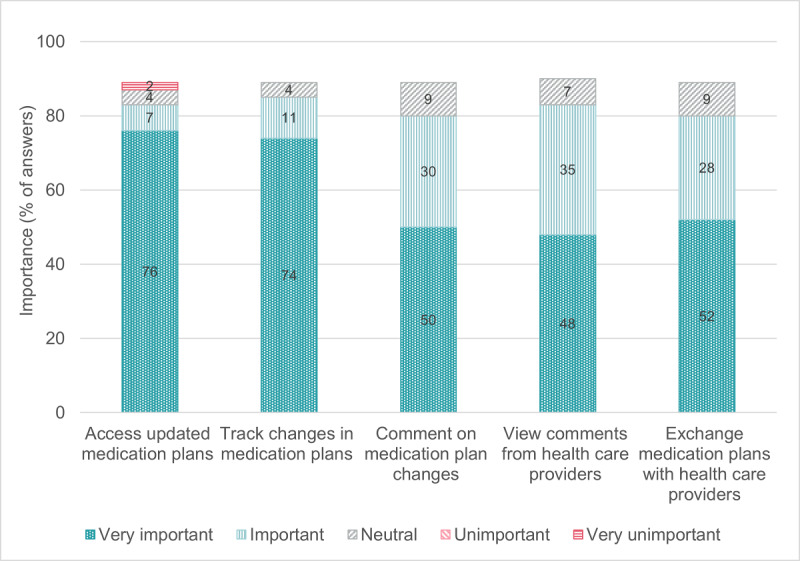
Primary care physician-reported importance of the functions in digital medication plan management tools (n=46). Missing data: 11.0% for all items presented in this figure.

**Figure 4.**
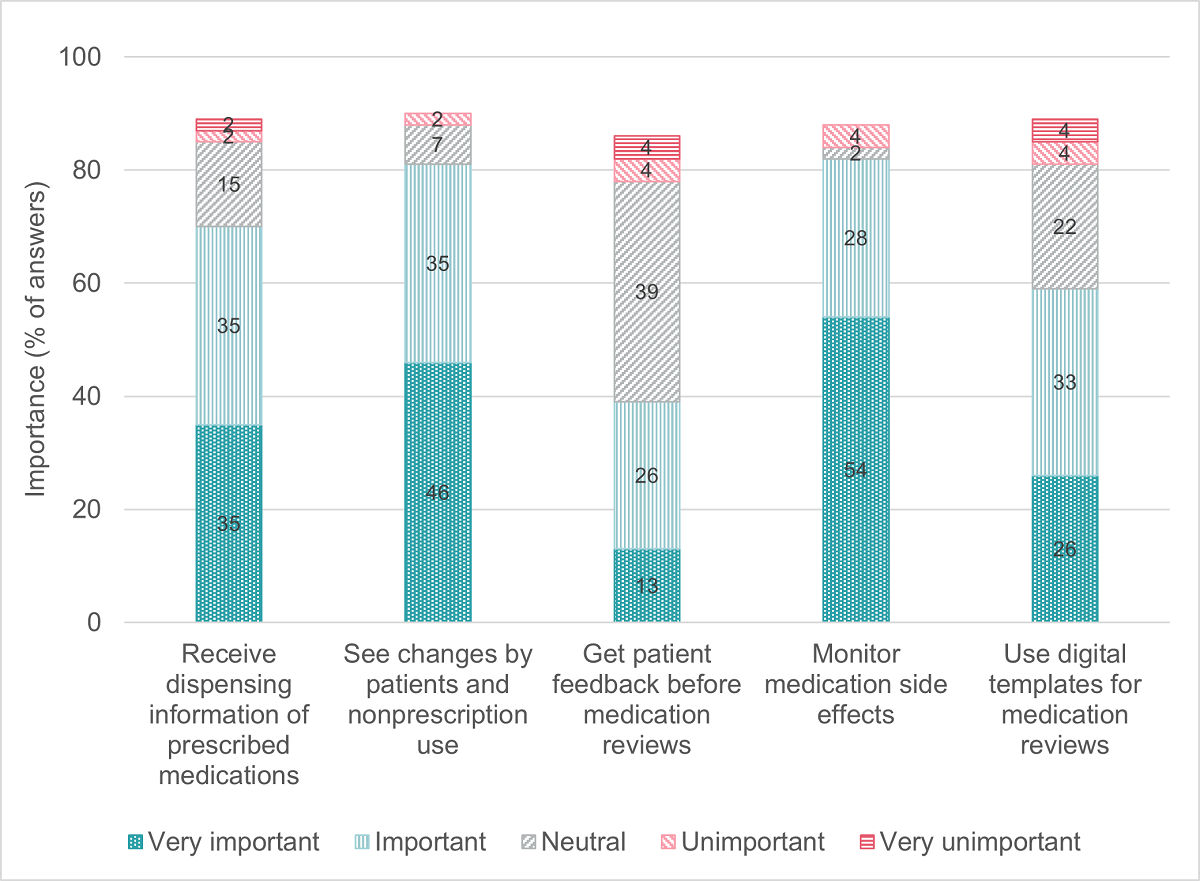
Primary care physician-reported importance of the functions in digital medication optimization tools (n=46). Missing data: 13.0% missing for “Get patient feedback before medication reviews”; 11.0% missing for all other items presented in this figure.

### Qualitative Semistructured Interviews

#### Participants

We interviewed 19 older adults (12/19, 63.2% female) with a mean age of 77 (SD 9) years and an average of 5 (SD 2) daily used prescription medications and 16 PCPs (5/16, 31.3% female) with an average professional experience of 11 (SD 7) years (Tables S4 and S5 in Multimedia Appendix 1). Our qualitative analysis identified several key themes relevant to both patients and PCPs, ranging from current use of medication management IT and barriers and enablers of current use of medication management IT to their willingness to adopt such tools in the future.

#### Current Use of Medication Management IT

Only a few older adults reported using medication management IT, namely smartphone apps with pill reminders, medication overviews, and/or documentations of medication intake. A lack of information about the existence of such tools, and the fact that health care providers did not mention them to patients, proved to be an important reason for patients not using them. All interviewed physicians reported using basic medication management IT integrated into their practice information systems by default, such as medication list managers, tools for generating and sending medication plans and prescriptions, and alerts for allergies and drug interactions. For conducting medication reviews, nearly all physicians relied on using drug interaction checkers within or outside their practice information system. Other medication management IT was rarely used for conducting medication reviews.

#### Willingness Regarding Future Use of Medication Management IT

##### Perspectives of Older Adults

Despite all older adults having at least one chronic condition and daily using medication (5 on average), most of them self-reported being in relatively good health. Most older adults felt confident to obtain and use their medication independently and perceived little need or benefit for IT supporting their medication management. However, many older adults considered medication management IT as useful, particularly for individuals with impaired cognition or complex medication regimens (eg, many different medications, irregular intake, or changing dosages). One older adult said:

Maybe I will have to take more medication when I get older, then maybe [medication IT] would help me.

[Male, 74]

Older adults believed medication management IT could enhance medication safety. They felt that tools like shared electronic medication plans or electronic patient records could improve information exchange among older adults, their relatives, and health care providers. One interviewee specified:

it is important that if you have to go to the hospital or if the [home care service comes to your house], that they know what [medication] to give you so that your medication intake is not interrupted.

[Female, 77]

The older adults considered features like updated medication overviews or pill reminders helpful to take medications correctly and on time. Several participants expressed concerns that medication management IT might be too complex and difficult to use. Therefore, many highlighted the importance of usability and accessibility (eg, simple programming, simple language, and large fonts) and usage support, ensuring accessibility for older adults and those less familiar with digital technologies. The motivation to use medication management IT also depended on the varying level of interest in and experience with digital technologies. One interviewee said:

Simply because I usually enjoy working with the computer. That could motivate me.

[Male, 85]

Many older adults doubted data protection in medication management IT, but this was of varying importance among the interviewees.

##### Perspectives of Primary Care Physicians

The physicians also underlined the importance of usability, accessibility, and efficiency in medication management IT to save time and facilitate work routines. However, concerns were raised about an increased workload or the need to alter established routines. The physicians preferred tools that are quickly and easily accessible, such as those integrated into or interoperable with the practice information system, to avoid entering data twice or making multiple clicks. One physician phrased it like this:

Sometimes it is just annoying because [the tool] is badly integrated and you have to click five times [to get where you want to] and it eats up your time.

[Male]

The physicians emphasized that medication management IT must provide patient-specific, clinically relevant information to support their current medication management and prevent medication-related errors. One physician formulated a negative example:

But if the digital tool tells me, “The best hypertension treatment according to the guideline is [this],” and I remember why I am doing it differently for this patient, this is of absolutely no use to me*.*

[Female]

They noted that frequent, nonrelevant alerts could lead to alert fatigue, where they might overlook important warnings due to the overwhelming number of notifications. When discussing an online tool providing access to a patient’s current medication for health care providers, patients, and their relatives, physicians noted it could enhance communication, collaboration, and patient involvement in medication optimization. But some feared that access by other health care providers could lead to loss of their oversight and the addition of inaccurate medications. One physician said

[...] what I do not want is that [...] without my knowledge […] [the medication list] is fiddled with and something is changed and I have nothing to say about it.

[Male]

Data protection concerns were raised, but some physicians did not view them as a sufficient reason to forgo the benefits of medication management IT. Additional medication management IT physicians wished to have in their practice information system were prescribing alerts for allergies, patient-specific risk factors (eg, poor kidney function) and medication shortages, reminder tools (eg, for conducting regular medication reviews), pharmacy dispensing information, and decision support tools integrated with patient data.

### Triangulation of Quantitative and Qualitative Data

After analyzing the quantitative and qualitative data separately, we triangulated the findings, using qualitative insights to contextualize and explain quantitative patterns (Table S3 in Multimedia Appendix 1). The data revealed that PCPs used a variety of medication management tools, although some tools were rarely adopted. Interviews clarified that PCPs primarily relied on integrated medication management IT within their practice information systems. However, they expressed frustration over missing features like embedded clinical decision support tools offering patient-specific recommendations based on the available electronic health data, personalized pop-up warnings, or reminder features. Older adults, in contrast, reported minimal use of such tools, which was corroborated by interviews indicating that most were unaware of the available options. While PCPs’ willingness to use IT varied depending on the tool, the most valued functionalities (eg, decision support tools) were often unavailable within their current IT infrastructure. Both PCPs and older adults consistently emphasized the importance of usable and accessible tools. Most older adults perceived medication management IT as potentially helpful, though some stated they would consider using it only if their medication regimen became more complex, their memory deteriorated, or if care partners helped them manage their medications. Interviews revealed that while most PCPs appreciated IT that can improve medication management and safety, some worried about unnecessary or misleading recommendations. A shared barrier across both groups was the perception that the effort required to use these tools might outweigh their benefits. Both older adults and PCPs appreciated the potential of medication management IT to enhance collaboration among patients, pharmacists, home care providers, and other health care professionals. Although opinions on data security diverged, with some voicing privacy concerns, others emphasized that the advantages of these technologies ultimately outweighed the associated risks.

## Discussion

### Principal Findings

Older adults rarely used medication management IT while PCPs reported regular use but, mainly limited to basic technologies integrated into their practice information systems, such as medication list managers and drug interaction checkers. Common barriers included a lack of information about available medication management IT, low perceived need and benefit, concerns about additional time and effort, expected complexity, and doubts about data protection. Nevertheless, most older adults and physicians had positive attitudes toward medication management IT, believing in their potential to enhance medication safety and facilitate medication management. They expressed willingness to adopt more medication management IT in the future if such technologies were easily accessible, usable, and supportive of their current medication management routines. We found no major discrepancies between physicians’ and patients’ views and expectations of medication management IT.

### Comparison With Prior Work

To our knowledge, this is the first study investigating the attitudes and expectations of both PCPs and patients regarding medication management IT in Swiss primary care settings. Our findings on barriers and facilitators for physicians’ use of medication management IT align with two reviews highlighting the importance of usability and accessibility, the belief in increased efficiency and quality of care, adequate support, appropriate recommendations, alert fatigue, and interoperability [48,49]. Even if the topic came up in our interviews, the need for training and financial incentives was given greater priority in the reviewed literature than in our findings [48,49]. While the literature on older adults’ attitudes toward using digital medication management IT is limited, the digital health literature shows barriers consistent with our findings, namely lack of knowledge about existing technologies, lack of information about their benefits, lack of self-efficacy in the use of such technologies, lack of support, and lack of user-friendliness [50]. In summary, despite differences in technologies and health care settings across countries, the literature highlighted similar key barriers and facilitators.

### Implications

We suggest developers, policy makers, and health care organizations in Switzerland and other countries collaborate more closely with health care providers and patients to develop and implement technologies that are tailored to their needs. To ensure older adults can access and benefit from medication management IT, it is crucial to address their specific needs, such as usability and accessibility (eg, large fonts and streamlined functionality), increase their awareness about these technologies, and provide appropriate support (eg, training and assistance from relatives or health care providers) [51-53]. Any training and assistance should consider the specific needs of older adults, including a slower learning pace, practical rather than theoretical content, and regular repetition [54]. In addition, a more proactive approach to raising awareness and user engagement is needed, as many PCPs and patients have limited knowledge about medication management IT, including its existence, how to access and use it, and its benefits. Given the low level of awareness among older adults, public health campaigns and health care professionals should inform patients about such tools in a way that is tailored to varying levels of digital literacy and interest, without underestimating older adults’ abilities [55]. Awareness campaigns could also target relatively healthy individuals to familiarize them with medication management IT early on. This may help ensure they are better prepared and more confident in using such tools once they become more frail and need to manage multiple medications. The digital divide among older adults is likely to shift in the future as more digitally adept generations grow older [56]. This could lead to higher demand for medication management IT in the future. An initial hurdle for health care providers to adopt collaborative technologies is that they are only beneficial if used by enough participants. Active promotion of medication management IT and creating incentives could encourage PCPs to use it [57,58]. Data protection emerged as a relevant concern in the interviews, reflecting a further challenge in the implementation of digital health technologies in many countries [59]. Data protection concerns negatively affect the willingness to use digital health services among patients [60]. To address these concerns, implementation strategies should include transparent communication about data use, as well as mechanisms that allow users to control which data are stored and shared [61,62]. Involving end users in the design and development process can help to ensure that privacy features align with their expectations and needs [61,62]. While Switzerland, as a high-income country with a federalist system, struggles with challenges related to a highly fragmented health care system with multiple practice information systems that differ across language regions, low-income countries face other challenges, such as lack of infrastructure, lack of insurance coverage, or lack of trained staff [63,64].

### Strengths and Limitations

A strength of this research lies in its mixed methods design, which enabled a deeper understanding of the gap between the generally positive attitudes and the limited use of medication management IT among older adults and PCPs in Swiss primary care settings. This approach not only explored the understudied topic of patients’ and providers’ perspectives in light of the recent roll-out of novel health technologies but also assessed specific examples of medication management IT, such as the eMediplan. Our findings can help developers, policy makers, and other stakeholders in the development, introduction, and optimization of medication management IT. This research also comes with several limitations. First, despite the convenience sampling of interviewees, we were able to achieve variability in patients’ age and living situation and physicians’ age and work experience, which might influence the adoption of medication management IT (eg, the younger among the older adults were more used to digital technologies). Nevertheless, the transferability of our findings is limited because our sample consisted of rather healthy older adults (eg, an average of “only” 4 or 5 medications, respectively), and the recruitment via an online research panel for the internet-based questionnaire led to a sample of digitally literate older adults, who can be hypothesized to have more favorable views toward medication management IT compared to their counterparts who are offline. Furthermore, participants who agreed to participate may have had an intrinsic interest in the topic of digital health. This may have led us to overestimate interest in digital medication management IT and the digital literacy of older adults in Switzerland. Despite this, the reported use of such tools among older adults was low, and the interviewed older adults strongly emphasized that usability and accessibility are also of central importance to them. However, it should be emphasized that individuals with limited digital competencies must not be excluded from future research on medication management supported by digital technologies. Second, despite diverse recruitment efforts, we were only able to recruit a small sample for the PCP questionnaire, limiting transferability and introducing potential selection bias. This may have led to an overestimation of PCPs’ actual use of and willingness to adopt digital technologies to support medication management. In addition, we were unable to calculate the response rate for the PCP questionnaire, since we could not assess how many physicians received the link to the internet-based questionnaire (eg, via newsletters). Third, in our interviews, we would have liked to explore medication-related functions of electronic patient records in greater depth; however, as only a minority reported using such tools, participant responses on this topic were limited or on a hypothetical basis. In contrast, PCPs’ greater familiarity with the Swiss Electronic Patient Record enabled a more in-depth discussion. Finally, to mitigate potential interviewer bias, we used standardized interview guides to minimize the influence of the interviewer’s perspective on participant responses.

### Conclusion

Information technologies are increasingly vital in health care, offering opportunities to improve medication management, optimization, and safety. Understanding user perspectives and needs is key to developing and sustainably implementing effective IT solutions in primary care. Our study emphasized the importance of accessible medication management technologies that enhance processes and safety without adding extra burden. Including perspectives from other health care providers, patients needing more assistance, and their care partners and assessing training needs for stakeholders to use such tools will generate evidence for implementing digital tools to improve medication safety in interprofessional health care settings.

## Supplementary material

10.2196/68857 Multimedia Appendix 1 Additional materials, figures, and tables supporting the study’s methodology and findings.

10.2196/68857 Checklist 1 COREQ checklist.

10.2196/68857 Checklist 2 CHERRIES checklist.

## Abbreviations

CHERRIES: Checklist for Reporting Results of Internet E-Surveys
COREQ: Consolidated Criteria for Reporting Qualitative Research
PCP: primary care physician
SSGIM: Swiss Society of General Internal Medicine
