# Supplementary material for: Using Medication Management Technologies in Swiss Primary Care: Mixed Methods Study
Source: J Med Internet Res. 2025 Aug 27;27:e68857. doi: 10.2196/68857 (PMC12385613; doi:10.2196/68857)
Supplement: Multimedia Appendix 1 [file jmir-v27-e68857-s001.docx]

**Supporting material:**

Using Medication Management Technologies in Swiss Primary Care: A Mixed Methods Study

Jeanne Maria Wildisen, MMed^1^; Alessia Romer, MSc^1,2^; Martina Zangger, MD^1,2^; Benjamin Bugnon, PhD^3^; Sven Streit, MD, PhD^1^; Kristie Rebecca Weir, PhD^1,4^; Katharina Tabea Jungo, PhD^1,5^

**Affiliations**

^1^ Institute of Primary Health Care (BIHAM), University of Berne, Bern, Switzerland

^2^ Graduate School for Health Sciences, University of Bern, Bern, Switzerland

^3^ Institute of Pharmaceutical Sciences of Western Switzerland, University of Geneva, Geneva, Switzerland.

^4^ Sydney School of Public Health, Faculty of Medicine and Health, The University of Sydney, Sydney, New South Wales, Australia.

^5^ Center for Healthcare Delivery Sciences (C4HDS), Division of Pharmacoepidemiology and Pharmacoeconomics, Department of Medicine, Brigham and Women's Hospital and Harvard Medical School, Boston, MA, USA.

**Table of content**

[Appendix 1: Questionnaire for online survey with older adults 3](#_Toc180650814)

[Appendix 2: Questionnaire for online survey with primary care physicians 8](#_Toc180650815)

[Appendix 3: Interview guide for interviews with older adults 16](#_Toc180650816)

[Appendix 4: Interview guide for interviews with primary care physicians 19](#_Toc180650817)

[Appendix 5: Cheat-sheet for the interviews with older adults and primary care physicians 21](#_Toc180650818)

[eTable 1: Codebook: Interviews with older adults 23](#_Toc180650819)

[eTable 2: Codebook: Interviews with primary care physicians 25](#_Toc180650820)

[eFigure 1: Recruitment flowchart: Online survey with older adults 27](#_Toc180650821)

[eFigure 2: Recruitment flowchart: Online survey with primary care physicians 27](#_Toc180650822)

[eTable 3: Baseline characteristics of interviewed older adults 28](#_Toc180650823)

[eTable 4: Baseline characteristics of interviewed primary care physicians 28](#_Toc180650824)

[eTable 5: Data triangulation 29](#_Toc180650825)

# **Appendix 1: Questionnaire for online survey with older adults**

*(Original version in German)*

**Dear participants,**

**Thank you for taking the time. The survey will take about 10 minutes.**

**We will ask you some questions about the management of your medication and about your experiences with and attitudes towards digital tools (e.g., Swiss Electronic Patient Record) in primary healthcare.**

**The survey is anonymous, and your data will be treated confidentially.**

**We thank you for your support.**

1. Do you regularly take two or more prescription medications?
   - Yes (required for survey participation)
   - No
   - I would not like to answer
2. How many prescription medications (pills, creams, active ingredient patches, etc.) do you take regularly?

TEXT FIELD (min. 2 required for survey participation)

1. In which year were you born?

TEXT FIELD (1924-1964 required for survey participation)

(Ineligible participants were sent to the end of the survey)

**Great, you belong to the target group of this survey. Let us get started!**

1. Which gender do you identify with?
   - Female
   - Male
   - Other gender

**Below we ask you some questions about the extent to which you are involved in managing your medications.**

1. Where do you get information from about your medication? (multiple answers possible)
   - In-person from healthcare professionals (e.g., physician, home care service provider, medical practice assistant)
   - In writing (e.g., brochure) from healthcare professionals (e.g., physician, home care service provider, medical practice assistant)
   - In-person from the pharmacy (e.g., pharmacists, pharmacy assistants)
   - In writing (e.g., brochure) from the pharmacy (e.g., pharmacists, pharmacy assistants)
   - Medication package leaflet
   - Internet, websites recommended by healthcare professionals/pharmacies
   - Internet, independently (e.g., search engine)
   - Relatives/acquaintances
   - Other

TEXT FIELD

1. How often does your primary care physician review and discuss your medication with you?
   - 3 x per year or more
   - 2 x per year
   - 1 x per year
   - less than 1 x per year
   - never
   - I do not know
2. What is your opinion on this frequency? My primary care physician reviews and discusses my medication with me ...
   - ... far too rarely
   - ... too rarely
   - ... at appropriate intervals
   - ... too often
   - ... far too often
3. To what extent do you agree with the following statements?

|  | Strongly disagree | Dis-agree | Agree | Strongly agree | I do not know |
| --- | --- | --- | --- | --- | --- |
| I trust my primary care physician. |  |  |  |  |  |
| My primary care physician and I meet as equals. |  |  |  |  |  |
| My primary care physician is professionally competent so he/she can recommend the best treatment for me. |  |  |  |  |  |
| I would like more information about the risks and benefits of my medication. |  |  |  |  |  |
| If I had more information about my medication, it would be easier for me to broach the subject with healthcare professionals. |  |  |  |  |  |
| If I had more information about my medication, it would be easier for me to help make decisions. |  |  |  |  |  |

**Next are a few questions about your medication list (= list of all your medications with dosages and times of intake) and any changes, such as starting new medications or reducing and stopping existing medications.**

1. Who should make decisions about your medication?
   - I should decide for myself which medication I take.
   - I should seek advice from my primary care physician so that I can make my own decisions about taking my medication.
   - My primary care physician and I should make decisions about my medication together.
   - My primary care physician should make decisions about my medication after listening to my opinion.
   - My primary care physician should make decisions about my medication.
2. How satisfied are you with the role you have in discussions with your primary care physician about changes to your medication list?
   - Very satisfied
   - Satisfied
   - Neutral
   - Dissatisfied
   - Very dissatisfied
3. Have you made any changes to your medication list independently in the last 6 months (e.g., adjusting the dose, stopping a medication, starting a new non-prescription medication)?
   - Yes, I have made changes on my own without informing my primary care physician.
   - Yes, I have made changes on my own and informed my primary care physician.
   - No, I have not made any independent changes
4. Do you receive a plan of your current medication from your primary care physician?
   - Yes, I receive an *eMediplan*. (Select this answer if you have an online or printed medication plan as shown in the picture)


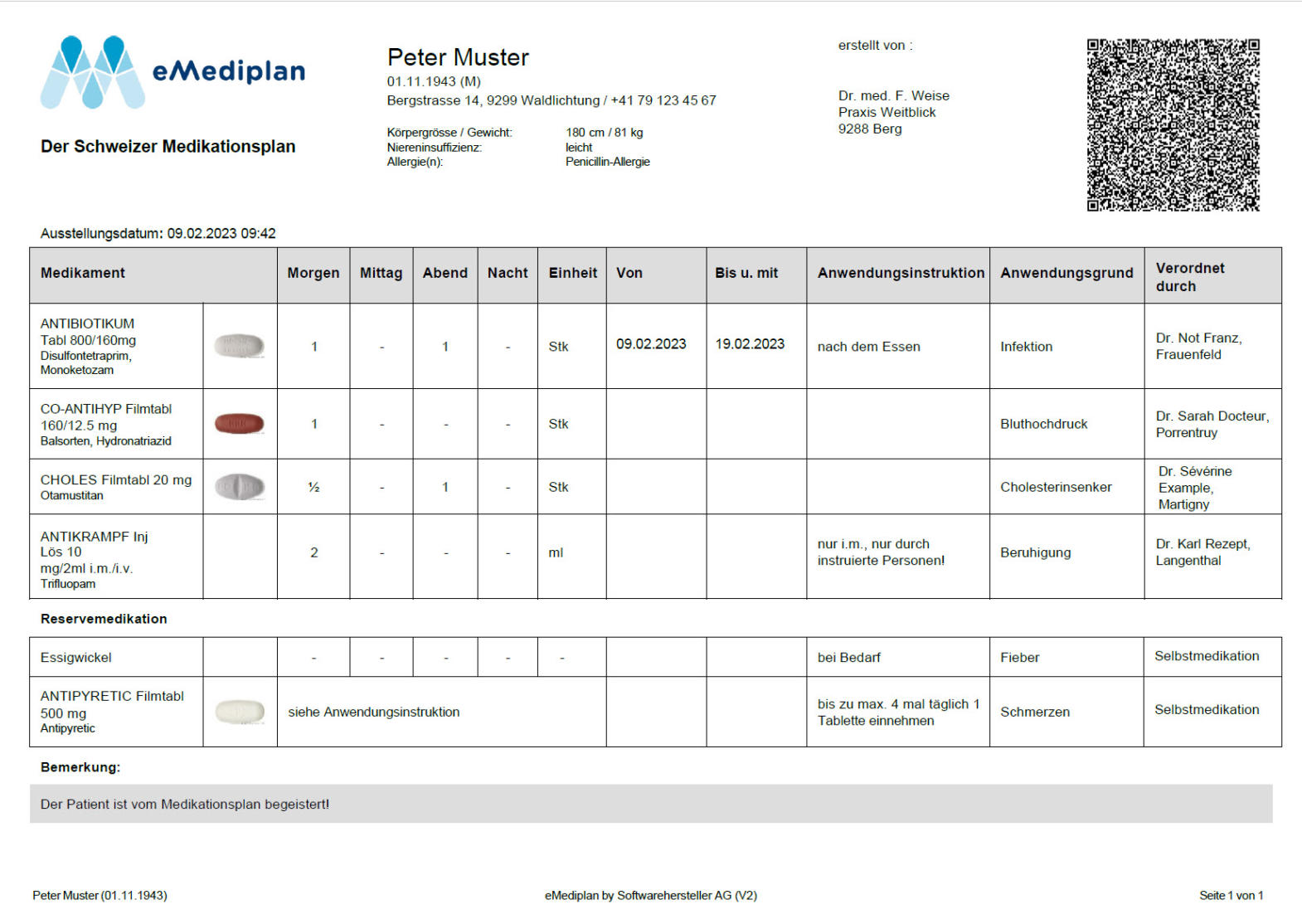
*https://emediplan.ch/bevoelkerung/details/*

- - Yes, I receive a different medication plan.
  - No, but I receive a medication plan from my pharmacy. (The label on your medication does not count as a medication plan)
  - No, but I have made my own medication plan.
  - No, I do not have a medication plan.

1. How satisfied are you with your medication plan?
   - Very satisfied
   - Satisfied
   - Neutral
   - Dissatisfied
   - Very dissatisfied
2. Is your medication plan always up to date?
   - Yes, me or a relative takes care of it.
   - Yes, my primary care physician takes care of it.
   - Yes, my pharmacy takes care of it.
   - No, but I know my current medication off by heart.
   - No, I often have difficulties taking my medication correctly because of this.
   - I do not know.
3. Would you like to receive a medication plan from your primary care physician?
   - Yes
   - No
   - I do not know
4. Have you started a new prescription medication in the last 6 months?
   - Yes
   - No
   - I would not like to specify
5. When the new medication was started, was it already discussed if and when this medication should be stopped again?
   - Yes
   - No

**In the next questions, we are interested in how confident you feel about handling medical data and new technologies.**

1. To what extent do you agree with the following statements?

|  | Strongly disagree | Disagree | Agree | Strongly agree | I do not know |
| --- | --- | --- | --- | --- | --- |
| I feel confident filling out medical documents (e.g., hospital registration form) |  |  |  |  |  |
| I often use digital devices (e.g. computer, smartphone, tablet). |  |  |  |  |  |
| I feel confident using most digital devices. |  |  |  |  |  |
| New ideas are needed in the field of digital technologies. |  |  |  |  |  |
| I keep up to date with new ideas in the field of digital technologies. |  |  |  |  |  |

**The Swiss Electronic Patient Record (EPR) is the online health record for the population in Switzerland. It is available on the Internet, belongs to you and contains health documents that are useful in the event of treatment. This information is stored by your healthcare professionals. You can also enter documents yourself, e.g., a copy of a report or your living will.**

1. Have you already opened an Electronic Patient Record?
   - Yes, I use an Electronic Patient Record.
   - Yes, but I do not use it.
   - No, but I am planning to open one in the future.
   - No, I did not know that existed.
   - No, I have not opened an Electronic Patient Record and am not planning to do so.
2. How satisfied are you with your Electronic Patient Record d?
   - Very satisfied
   - Satisfied
   - Neutral
   - Dissatisfied
   - Very dissatisfied
3. To what extent do you agree with the following statements?

|  | Strongly disagree | Disagree | Agree | Strongly agree | I do not know |
| --- | --- | --- | --- | --- | --- |
| The Electronic Patient Record serves to improve efficiency in the healthcare system. |  |  |  |  |  |
| The Electronic Patient Record serves to improve the quality of patient care in the healthcare system. |  |  |  |  |  |
| The Electronic Patient Record helps me to better understand my own health. |  |  |  |  |  |
| The Electronic Patient Record stores my health data securely. |  |  |  |  |  |

1. To what extent do you agree with the following statements?

|  | Strongly disagree | Disagree | Agree | Strongly agree | I do not know |
| --- | --- | --- | --- | --- | --- |
| I would like to use an Electronic Patient Record to manage my own health information. |  |  |  |  |  |
| I would like to use an Electronic Patient Record to share my own health information with healthcare professionals. |  |  |  |  |  |
| The Electronic Patient Record serves to improve efficiency in the healthcare system. |  |  |  |  |  |
| The Electronic Patient Record serves to improve the quality of patient care in the healthcare system. |  |  |  |  |  |
| The Electronic Patient Record would help me to better understand my own health. |  |  |  |  |  |
| The Electronic Patient Record would store my health data securely. |  |  |  |  |  |

**Stopping or reducing medication may be considered in consultation with your physician in situations where the medication is no longer needed, or the potential harm outweighs the benefit. We would like to ask you some questions about this in the following section.**

1. How important do you find the following statements: Stopping or reducing unnecessary and/or high-risk medication helps ...

|  | very  Unimportant | Unimportant | Neutral | Important | Very  important |
| --- | --- | --- | --- | --- | --- |
| ... to improve the quality of life of patients. |  |  |  |  |  |
| ... to prevent undesirable side effects in patients. |  |  |  |  |  |
| ... to reduce the treatment burden on patients. |  |  |  |  |  |
| ... to increase sustainability in the healthcare sector. |  |  |  |  |  |
| ... to distribute the available resources in the healthcare system more fairly. |  |  |  |  |  |
| ... to reduce healthcare costs. |  |  |  |  |  |

**We have reached the end of the survey.**

**Thank you very much for your participation!**

**Click on the "Done" button to send the survey.**

# **Appendix 2: Questionnaire for online survey with primary care physicians**

*(Original version in German)*

**Thank you for your interest!**

**The survey is available in three languages: German, French and Italian. You can select the language that suits you best (top right).**

Dear colleagues,

Our survey relates to the use of electronic information and communication technology in healthcare (digital health or eHealth). We want to know how often you use digital tools and

whether you would be willing to use them more. Your honest answers will help us understand what physicians like you need when using digital tools in clinical work processes.

Important information about the survey:

- The survey is anonymous, and all data will be treated confidentially.
- The survey takes 15 to 20 minutes and contains between 38 and 47 questions. Depending on the answer, certain questions are automatically skipped.
- If you are completing the survey on your mobile phone, please note that some questions are not very clearly arranged. To reach the "Next" button, you have to scroll down.

If you agree to participate, please click on the "Continue" button.

Thank you for your support!

Inclusion criteria

**Before we begin, we would like to make sure that you belong to the target group of this survey. Please answer the following questions.**

1. Do you practice general internal medicine in Swiss primary care?
   1. Yes
   2. No (End of participation)
2. Do you have a specialist title in general internal medicine?
   1. Yes
   2. No
   3. Not yet, I am in further training at the moment
3. Do you have another specialist title in addition to general internal medicine?
   1. Yes
   2. No

If yes, what other specialist title do you have?

TEXT FIELD

Socio-demographic and geographical information

**In this section we would like to learn more about your individual situation in everyday practice.**

1. In which canton do you practice?

DROPDOWN

Zurich (ZH)

Berne (BE)

Lucerne (LU)

Uri (UR)

Schwyz (SZ)

Obwalden (OW)

Nidwalden (NW)

Glarus (GL)

Zug (ZG)

Fribourg (FR)

Solothurn (SO)

Basel-City (BS)

Basel-Country (BL)

Schaffhausen (SH)

Appenzell Outer Rhoden (AR)

Appenzell Inner Rhoden (AI)

St. Gall (SG)

Grisons (GR)

Aargau (AG)

Thurgau (TG)

Ticino (TI)

Vaud (VD)

Valais (VS)

Neuchâtel (NE)

Geneva (GE)

Jura (JU)

1. Where is the primary care practice you work in located? If you work in more than one of them, select the one you spend most of the time working in.
   1. Urban area
   2. Suburban area
   3. Rural area
2. What is your gender identity?
   1. Female
   2. Male
   3. Non-binary
   4. I would not like to answer
3. How old are you (in number of years)?

TEXT FIELD

1. Have you completed your further training in Switzerland, or are you in the process of completing your further training in Switzerland?
   1. Yes
   2. No
   3. Partial
2. How many years have you been practicing general internal medicine in primary care?

TEXT FIELD

1. What is the most spoken language in your everyday practice?
   1. German/Swiss German
   2. French
   3. Italian
   4. Other (please specify)

TEXT FIELD

1. Are you employed or self-employed?
   1. Employed
   2. Self-employed
   3. Other (please specify)

TEXT FIELD

1. What kind of practice do you work in?
   1. Group practice
   2. Individual practice
   3. Outpatient center
   4. Other type of practice (please specify)

TEXT FIELD

1. Is the practice where you work part of a network (e.g., medbase, mediX etc.)?
   1. Yes
   2. No
2. How many primary care physicians work in your practice (including you)?
   1. I do not know
   2. Please enter your answer here.

TEXT FIELD

1. How many consultations do you have on average per half working day? If you do not know exactly, please estimate.

TEXT FIELD

1. How many half-days a week do you normally have consultations with patients?

TEXT FIELD

General questions on the use of digital technologies in clinical work

**In this section, we would like to find out how you use digital technologies in everyday practice.**

1. How would you rate your skills in using digital devices (e.g., PC, smartphone, tablet)?
   1. Very poor
   2. Poor
   3. Neither good nor poor
   4. Good
   5. Very good
2. Do you use electronic medical records in your practice?
   1. Yes, the patients’ medical records are completely electronic.
   2. Partly, the patients’ medical records are partly electronic.
   3. No, patients’ medical records are kept exclusively on paper.
3. How long have you been using a practice information system in your clinical practice? (in years)
   1. I do not know
   2. Please enter your answer.

TEXT FIELD

1. What types of communication and activities does your practice information system allow?
   1. Secure e-mails or communication with other professionals (e.g., HIN, TrustID)
   2. Secure messaging to patients (no e-mail)
   3. Documentation of patient treatments
   4. Creation of diagnostic lists
   5. Electronic prescriptions, issuing prescriptions (e.g., with QR code)
   6. Documentation of vital data
   7. Creation of medication lists
   8. Creation of medication plans
   9. Referral management to specialists
   10. Receiving lab requests and results
   11. Online appointment booking for patients
   12. Billing
   13. Other (please specify)

TEXT FIELD

1. How often do you ask your patients if they use an electronic device or app to monitor their health?
   1. Always
   2. Very often
   3. Sometimes
   4. Rarely
   5. Never
2. Are you connected to an Electronic Patient Record system (e.g., CARA, eSANITA, Sanela, etc.)?
   1. Yes
   2. No

Willingness to use new digital technologies and digital health interventions

**In this section, we would like to learn more about your readiness to use new digital technologies and digital health interventions in clinical practice. By the term "digital health interventions" we mean all electronic health services such as telemedicine, electronic health records and health apps (mHealth) with and without remote monitoring.**

1. How much do you agree with the following statements

|  | Strongly agree | Somewhat agree | Neither agree nor  disagree | Somewhat  disagree | Strongly disagree |
| --- | --- | --- | --- | --- | --- |
| I have an idea of what digital health  interventions are. |  |  |  |  |  |
| I have some knowledge of how  digital health interventions work. |  |  |  |  |  |
| I could imagine incorporating digital  health interventions into my work. |  |  |  |  |  |
| I intend to try out digital health interventions in my work within the  next 3 months. |  |  |  |  |  |
| The technical equipment of the practice is sufficient for the implementation of digital health interventions (e.g., sufficient computers, fast internet, security, etc.). |  |  |  |  |  |

1. How much do you agree with the following statements

|  | Strongly agree | Somewhat agree | Neither agree nor  Disagree | Somewhat  Disagree | Strongly disagree |
| --- | --- | --- | --- | --- | --- |
| I would easily learn to work with a digital health intervention. |  |  |  |  |  |
| My colleagues would support the  use of digital health interventions. |  |  |  |  |  |
| Our patients would support the use  of digital health interventions. |  |  |  |  |  |
| Digital health interventions could  improve the health of patients. |  |  |  |  |  |
| Digital health interventions would be a useful addition to existing  treatment. |  |  |  |  |  |

Randomization of the specific case studies

**To make it easier for you to answer the survey, the questions are randomized into specific case studies. Please answer this question.**

1. Is your birthday an even or odd number? (Randomization)
   1. Even
   2. Odd

Use of digital technologies: Use to optimize medication prescriptions

**The aim of this part of the survey is to determine your attitude towards conducting medication reviews and the use of digital tools for this purpose.**

**By *medication review*, we mean any structured process that is carried out to review patients' medication with the aim of optimizing medication intake and improving health outcomes.**

**By *medication list* we mean an overview of previous and current medication. It contains current and previous information on treatment decisions, prescriptions and dispensing of medication.**

**By *medication plan* we mean an overview as complete as possible of the current medication (extract from the medication list on the current date).**

1. Do you provide your patients with 5 or more medications with a medication plan (on paper or digitally) in addition to the prescriptions?
   1. Never
   2. Rarely
   3. Sometimes
   4. Often
   5. Very often
2. Can you easily print out a medication plan with your practice information system that summarizes the medication and the most important instructions for taking medication and make it available to patients?
   1. Yes
   2. No
3. Do you use the *eMediplan* in your practice?
   1. Yes
   2. No
4. Why do you not use the *eMediplan*?
   1. I do not want to use the *eMediplan*
   2. I do not use the *eMediplan* yet but would like to use it in the future.
   3. I cannot use the *eMediplan* for technical reasons
   4. Other reason

TEXT FIELD

1. How often do you encourage your patients to bring a medication plan with them when they visit you and/or other healthcare professionals?
   1. Never
   2. Rarely
   3. Sometimes
   4. Often
   5. Very often
2. Briefly describe how often you check the medication lists of your patients with more than 5 medications.

TEXT FIELD

1. When you think about how often you check the medication lists of your patients with more than 5 medications: What are your thoughts on the frequency of medication

reviews?

- 1. I do it far too often (frequency far too high)
  2. I do it too often (frequency too high)
  3. The frequency is just right
  4. I do it too rarely (frequency too low)
  5. I do it far too rarely (frequency far too low)

Please read the following quality indicator

**The Swiss Society of General Internal Medicine (SGAIM) has published the following quality indicator: "Recognize medication interactions and prevent side effects: Proportion of patients aged 65 years or older taking at least 5 long-term medications who have had a medication review with interaction check in the last 12 months. The aim of the indicator is to record and prevent potential medication interactions and side effects." Quality indicators in the outpatient sector no.3**

1. Have you already heard of this quality indicator?
   1. Yes
   2. No
2. How useful do you find this quality indicator for your clinical practice?
   1. Not useful at all
   2. Not useful
   3. Neutral
   4. Useful
   5. Very useful
3. How often do you think you implement this quality indicator in your practice?
   1. Never
   2. Rarely
   3. Sometimes
   4. Often
   5. Very often
4. Why do you think you conduct a medication review of patients with polypharmacy less than once a year?

TEXT FIELD

Tools for conducting medication reviews

1. Do you use tools that support you in medication optimization?
   1. Yes
   2. No
2. What tools do you use when conducting medication reviews? Select all the answers that apply.
   1. Interaction checker integrated into the practice information system
   2. Interaction checker outside the practice information system (e.g., CDS)
   3. Electronic decision aids
   4. Guidelines
   5. Lists for recognizing/assessing potentially inappropriate medications (e.g., Priscus list, Beers criteria)
   6. Online evidence-based clinical resources (e.g., UpToDate)
   7. Structured forms for conducting medication review (paper)
   8. Structured forms for conducting medication review (digital)
   9. Apps
   10. Tools that can reconcile different medication lists
   11. Other tools (please specify)

TEXT FIELD

1. After conducting a medication review and in the case of planned medication changes, how do you document these changes?
   1. Medication list is adjusted
   2. Comment as free text in the patient record
   3. Other form of documentation (please specify)

TEXT FIELD

1. After conducting a medication review and in the event of planned medication changes, how do you document and communicate these changes to your patients and/or their relatives? Select all the answers that apply.
   1. Verbal information on change(s)
   2. Written information on change(s)
   3. Print and hand over a new prescription
   4. Print and hand out adapted medication plan
   5. Other form of communication (please specify)

TEXT FIELD

1. After conducting a medication review, do you communicate the changes with other healthcare professionals?
   1. Yes
   2. No
2. If yes, with which ones? Select all the answers that apply.
   1. With pharmacists
   2. With other physicians
   3. With nursing staff
   4. With other health professionals
3. Would you be willing to hand over or delegate the conduct of a medication review to another healthcare professional (e.g., pharmacist)?
   1. Yes
   2. Rather yes
   3. Neutral
   4. Rather no
   5. No
4. The provider of the practice information system I work with already offers an integrated digital tool for medication optimization, which is more than just an interaction checker.
   1. Yes
   2. No
5. I would like the provider of my practice information system to offer a digital tool for medication optimization integrated into the practice information system.
   1. Yes
   2. No

Evaluation of possible functions for medication lists and reviews

1. How important would you find the following functions in a digital tool that supports you in managing your patients' medication list and conducting medication reviews? Indicate the importance of the following answer options.

|  | Very important | Important | Neither important nor unimportant | Unimportant | Very unimportant |
| --- | --- | --- | --- | --- | --- |
| Access to the current and complete  medication plan that has been updated by me and/or other healthcare professionals (e.g., in the outpatient, inpatient setting, etc.). |  |  |  |  |  |
| Tracking changes in patients' medication plans (starting, stopping, dose adjustments, etc.). |  |  |  |  |  |
| Preparation of a summary of the most important changes with a comparison at admission and discharge in the event of hospitalization. |  |  |  |  |  |
| The ability to comment on changes I have made to the medication plan. |  |  |  |  |  |
| The ability to read the comments of other healthcare professionals who have made changes to the medication plan. |  |  |  |  |  |
| Electronic exchange of medication plans to ensure the transfer of information to other healthcare professionals (e.g., home care, hospital, pharmacy). |  |  |  |  |  |
| Receiving information on the dispensing of the medication prescribed by me. |  |  |  |  |  |
| Insight into the changes made by patients and the use of non-prescription medication. |  |  |  |  |  |
| Obtain patient feedback before conducting a medication review (e.g., structured short questionnaire). |  |  |  |  |  |
| Insight into the instructions given to patients by the pharmacist and possible adaptation of these instructions |  |  |  |  |  |
| Provision of targeted information to support the self-management of patients and relatives. |  |  |  |  |  |
| Recording and monitoring of side effects caused by taking medication |  |  |  |  |  |
| Integration of digital, structured forms for conducting and documenting medication reviews. |  |  |  |  |  |
| Tools that can reconcile different medication lists. |  |  |  |  |  |

1. How much do you agree with the following statements?

|  | Strongly agree | Somewhat agree | Neither agree nor  disagree | Somewhat  disagree | Strongly disagree |
| --- | --- | --- | --- | --- | --- |
| If available, I would like to use a digital tool for medication optimization that is integrated into my practice information system (e.g., reminder for medication review). |  |  |  |  |  |
| If available, I would like to use a digital platform to coordinate medication optimization with my patients and other healthcare professionals (e.g., pharmacists, home care service, etc.). |  |  |  |  |  |

End of the survey. Thank you very much for your participation!

**We really appreciate the time you have taken to complete this survey.**

**Please click on the ‘Done’ button.**

# **Appendix 3: Interview guide for interviews with older adults**

*(Original version in German)*

1. **Introduction**

- Interviewer introduction
- Study introduction: Topic of the questions, aims of the study, inclusion/exclusion criteria, relevance
- Hand out cheat sheet with definitions to read and refer to later in the interview
- Explain that the study is anonymous and confidential and that questions can be skipped, or the interview can be cancelled at any time
- Obtain consent for audio recording
- Thank the interviewee

1. **General questions**

- What is your daily routine for taking medication?
  1. Probe: How many medications do you take regularly?
- What goes through your mind when you think of digital technologies?

1. **Involvement in the use of medication**

- To what extent are you responsible for your own medication (obtaining prescriptions, ordering/collecting medication, preparing medication, taking medication on time and correctly)?
  1. Probe: Do you need help with any of this?
  2. Probe: Who helps you with this?
- What role do you have in discussions about your medication with your primary care physician?
  1. How satisfied are you with this role?
  2. What information do you receive during such a discussion?
     1. Does this information help you to participate in decisions about your medication?
     2. Where else do you get information about your medication?
- How would you rate your knowledge about your medication? (benefits, risks, side effects)
  1. In your opinion, is this knowledge sufficient?
- Have you ever made any changes to your prescribed medication yourself?
  1. What kind of change?
  2. Have you told your primary care physician about this?

1. **Digital (health) technologies**

- How well do you use computers, mobile phones etc.?

Probes:

| if rather well | if not very well/not at all |
| --- | --- |
| How did you learn this? | What would make it easier for you to use? |
| To what extent do digital technologies make your everyday life easier? | Can someone help you if you have problems? How do you feel about this? |

*Let's take a look at our cheat sheet together for the definition of digital health technologies and digital tools for medication management.*

- What goes through your mind when you read the information about these digital health technologies and digital tools for medication management?
- What experiences have you had with such digital health technologies or what have you heard about them?
- What digital tools do you use to manage your medication?
  1. *If you are not familiar with these tools:* Can you explain how they work?
  2. What do you like about them?
  3. What do you dislike about them?
  4. What motivated you to use this/these tool(s)?
- What do you think digital tools are generally useful for in managing your medication?
- What expectations do you have of digital tools for the management of your medication?
- What prevents you from using such tools?
  1. What could help you overcome these barriers?
- What would motivate you to use (other) digital tools for your medication management?
- Do you think your health data are sufficiently protected when using digital health technologies?

1. **Medication plan and shared electronic medication plan**

*Let us take another look at our cheat sheet for the definition of medication plan and shared electronic medication plan (e.g., eMediplan).*

- What goes through your mind?
- Do you have a medication plan yourself?

| Yes | No |
| --- | --- |
| Where did you get this plan? | For what purposes could a plan like this be useful for you? |
| What information does the plan contain? |  |
| In what form do you use the plan? Digitally? On paper? |  |
| What do you use your (electronic) medication plan for (e.g., help with taking medication, take along to consultations)? |  |
| Is your (electronic) medication plan always kept up to date?   - Probe: How? |  |
| How satisfied are you with your (electronic) medication plan?   - Probe: What do you like about it? - Probe: What don’t you like about it? |  |

- Could you please tell me about your experience with the *eMediplan* or what you know about it? (*show an example*)
- What is your attitude towards a shared electronic medication plan?
- In your opinion, what is the purpose of using a shared electronic medication plan?
  1. How does/could a shared electronic medication plan help you with your medications?
- For what reasons do you (not) use a shared electronic medication plan?
  1. *No shared electronic medication* *plan available*: What would have to change to make you want to use a shared electronic medication plan?
- Who should have access to which information in a shared electronic medication plan?
  1. What information should be made available in an emergency?
- Who should keep a shared electronic medication plan up to date? (primary care physician, other physicians, nursing staff, pharmacist, yourself, relatives, ...)

1. **Electronic Patient Record (EPR)**

*Let us take another look at our cheat sheet for the definition of the Electronic Patient Record (EPR).*

- What goes through your mind?
- Could you please tell me about your experiences with the Electronic Patient Record or what you know about it?
- How do you see the benefits of an Electronic Patient Record?
- Have you already opened an Electronic Patient Record?
  1. *If yes:* Why have you opened an Electronic Patient Record?
  2. *If yes:* What do you use the Electronic Patient Record for?
  3. *If yes:* To what extent does the Electronic Patient Record help you with your medication use?
  4. *If yes:* What do you like about it?
  5. *If yes:* What do you dislike about it?
  6. *If* *no:* Why have you not yet opened an Electronic Patient Record?
  7. *If* *no:* All your medication can be stored and processed in the Electronic Patient Record. How could the Electronic Patient Record help you with your medications?

1. **Other digital tools for medication management**

- If you could wish for another digital tool for medication management such as a smartphone app, electronic platform, online information, electronic device, etc., what would this program look like/what functions would it have?

1. **Conclusion**

- Do you have anything else to say on the topic that we have not yet discussed?
- Do you know anyone else who would be interested in taking part in an interview?
- Contact details + age + living situation

# **Appendix 4: Interview guide for interviews with primary care physicians**

*(Original version in German)*

1. **Introduction**

- Interviewer introduction
- Study introduction: Topic of the questions, aims of the study, inclusion/exclusion criteria, relevance
- Hand out cheat sheet with definitions to read and refer to later in the interview
- Explain that the study is anonymous and confidential and that questions can be skipped, or the interview can be cancelled at any time
- Obtain consent for audio recording
- Thank the interviewee

1. **General questions**

- Could you briefly introduce yourself and your daily work routine?
  1. Probe: Since when have you been a primary care physician?
  2. Probe: What kind of practice do you work in?
  3. How many people work with you in the practice?
  4. Where is the practice located?

1. **Digital technologies in daily practice**

- Which practice information system do you use in your practice? (*Cheat sheet only if needed*)
  1. What do you like about it? What do you dislike about it?
  2. What tools does the practice information system offer for managing and optimizing medications?
     1. To what extent do you use these tools in your daily practice?
     2. What do these tools help you most with?
- Are you connected to an Electronic Patient Record? (*Cheat sheet only if needed*)
  1. What do you like about it? What do you dislike about it?

1. **Medication plan and shared electronic medication plan**

*Let us take another look at our cheat sheet for the definitions of the medication plan and shared electronic medication plan.*

- Can you describe how and when you prepare medication plans?
- Are the plans available digitally and/or printed?
  1. *If digital:* Can you tell me more about the software (e.g., *eMediplan*) you use for this?
     1. Probe: Do you prepare *eMediplans* in your practice?
     2. Probe: Is this software integrated into your practice management system?
  2. *If digital:* Who has access to them and who can edit them?
- How and by whom are the medication plans kept up to date?
  1. In your opinion, who is responsible for regularly reviewing and updating a medication plan if necessary?
- What is your attitude towards shared electronic medication plans?
  1. What information is useful in a shared electronic medication plan?
  2. Probe: In your opinion, what are the advantages and disadvantages of shared electronic medication plans?

1. What could be improved?
   1. *If rather negative:* What are the reasons for your cautious attitude?

- What motivates you to use a shared electronic medication plan or what prevents you from doing so?
- What tasks should patients, physicians, pharmacists, and other healthcare professionals have in managing and updating the *eMediplan*?
- What do you think would be necessary for a nationwide implementation of the *eMediplan* so that both primary care physicians and patients could benefit from it?
- What goes through your mind when you hear that the use of the *eMediplan* is supposed to become mandatory for all physicians?

1. **Medication reviews and digital tools for medication reviews**

*Let us take another look at our cheat sheet for the definitions of medication reviews and digital tools for medication reviews.*

- Please briefly describe how you conduct medication reviews.
  1. Probe: How often does this happen?
  2. Probe: What tools do you use for this?

1. What role do digital tools play to support medication reviews?
   1. How do you document medication changes?

- What helps you to regularly conduct medication reviews?
- What prevents you from regularly conducting medication reviews?
- To what extent do you involve your patients in the medication optimization process?
  1. Could you imagine involving patients more?
     1. *If yes:* How?
     2. *If* *no:* Why not?
- What is your attitude towards digital tools for medication reviews?
  1. Probe: In your opinion, what are the advantages and disadvantages of digital tools for medication reviews?
  2. *If* *rather negative:* What are the reasons for your rather negative attitude?
- What motivates you, or would motivate you, to use digital tools for medication reviews?
- What prevents you from using digital tools for medication reviews?
- What features do digital tools for medication reviews need to offer to be helpful in your daily practice routine?

1. **Other tools for medication management**

*Let us take another look at our cheat sheet for the definitions of digital health technologies and digital tools for medication management.*

- If you could wish for a digital tool for managing and optimizing medication, what would it look like/what functions would it have?
- What prevents you from using new digital tools for medication management in your daily practice?
- What motivates you to use new digital tools for medication management in your daily practice?

1. **Closure**

- Is there anything else you would like to say on the topic that we have not discussed yet?
- Do you know anyone else who might be interested in participating in an interview (patients or primary care physicians)?

# **Appendix 5: Cheat-sheet for the interviews with older adults and primary care physicians**

*(Original version in German)*

| **Term** | **Explanation** |
| --- | --- |
| **Digital health technologies (eHealth technologies)** | Technologies in the healthcare system such as Electronic Patient Records, medical telephone counselling, health apps, and wearable devices (e.g., fitness watches). Digital health technologies aim to improve efficiency, quality, accessibility, and communication in the healthcare system. |
| **Digital aids/technologies for medication management** | Digital health technologies developed specifically for the management and optimization of patients' medication. Examples include the *eMediplan* (shared electronic medication plan), medication apps, reminders to take medication on devices, platforms with medication information. |
| **Medication plan** | List of current medication that a person is taking. The list contains the name, dosage, time of intake, instructions for taking the medication and other information about each medication.  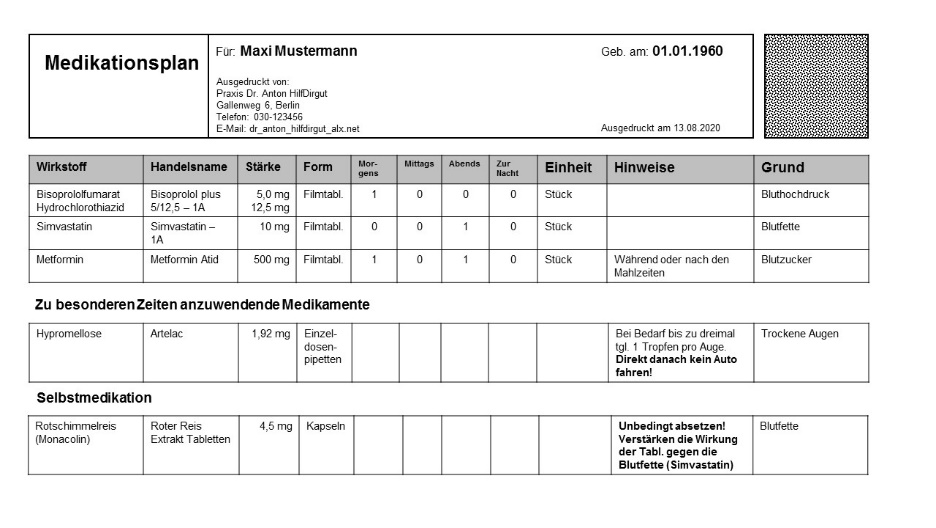  *https://www.barmer.de/gesundheit-verstehen/medizin/medikamente/wofuer-medikationsplan-1056422* |
| **Shared electronic medication plan (e.g., *eMediplan*)** | Medication plan that can be read, updated, and given to patients by the healthcare provider in charge via QR code. Its purpose is to simplify communication, keep the medication plan up to date and create an overview and clarity for patients.  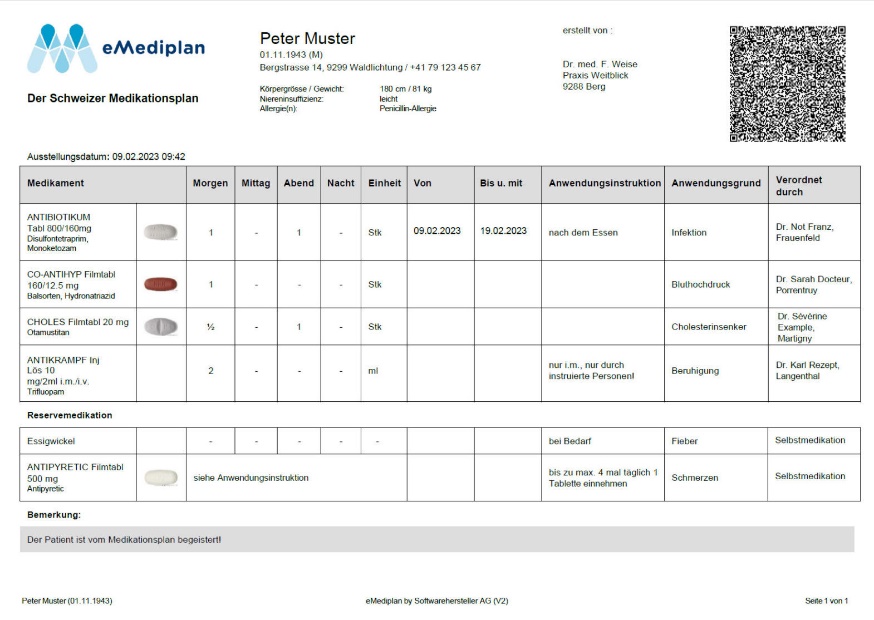  *https://emediplan.ch/bevoelkerung/details/* |
| **Electronic Patient Record (EPR)** | Digital version of the patient record that belongs to the patient. It contains health information and documents such as medical reports, nursing reports, X-ray findings, laboratory results, vaccination records, medication lists and information on allergies.  The aim of the EPR is the secure exchange of health information between patients and healthcare professionals for high-quality patient care.  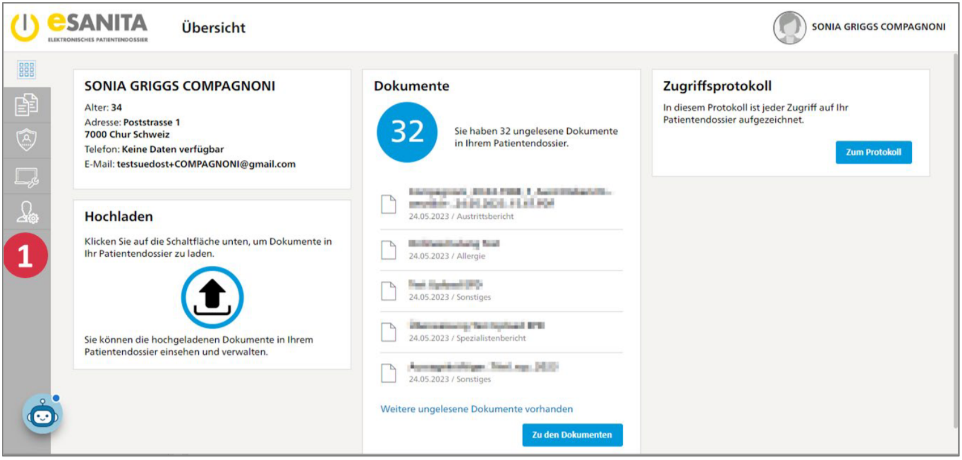*Verein eSANITA – 13180 EPD-Patientenanleitung / Version 1.4 / 3. Juni 2024* |
| **Medication review** | Systematic review and assessment of medication by a healthcare professional in collaboration with the patient. The aim of a medication review is to ensure the safety and effectiveness of the medication: adjusting dosages, avoiding interactions, reducing side effects, and taking individual needs into account. |
| **Digital tools for medication reviews** | Digital technologies that support regular, high-quality medication reviews. Examples are interaction checkers, electronic medication review reminders, clinical decision support systems (combines patient data and medical expertise to generate treatment recommendations). |
| **Practice information system** | Software for managing and organizing clinical and administrative processes in medical offices. Functions may include management of patient data, medical documentation (patient files), appointment scheduling, billing, and team communication. |

# **eTable 1: Codebook: Interviews with older adults**

*(Original version in German)*

| **Code** | **Subcode** | **Definition of (Sub)code** |
| --- | --- | --- |
| Digital technologies | Attitude | Description of participants' opinions on digital technologies (e.g., phone, computer, tablet). Positive and negative aspects of digital technologies. Description of how digital technologies are helpful or a nuisance in daily life. Technologies that are used or not used in daily life. |
|  | Difficulties | Statements where someone struggles with digital technologies like computers, tablets, or phones, and possible reasons for it. Situations in which participants encounter problems and/or need help. |
|  | Facilitators | Statements where someone is comfortable using digital technologies like computers, tablets, or phones, and possible reasons for that. Things that make the use of digital technologies easier. |
| Medication | Number | Number of prescribed and over-the-counter medications (daily + irregularly). |
|  | Intake | Routine of daily medication intake. |
|  | Procurement | Routine of obtaining medications (getting prescriptions, picking up/receiving medications). |
|  | Difficulties | Difficulties in daily medication intake. Reasons why medications are forgotten or not taken. Other issues with medication management (e.g., getting prescriptions, picking up/receiving medications, remembering names, etc.). |
| Medication information | Knowledge | Knowledge about one's medications. Facts mentioned about the medications during the interview and statements about participants’ self-assessment of their knowledge about their medications. |
|  | Sources | Participants' sources of medication information. |
|  | Attitude | Statements about how important information about their own medications is to the participants. Positive and negative aspects of information about their own medications or specific sources of information. |
| Medication decision | Process | The process by which the current medication regimen was decided. Attitudes toward their own medications. |
|  | Relationship | Primary care physician-patient relationship. The patient’s role in medication decisions. How the relationship with the physician influences medication intake. The extent to which patients want to participate in medication decisions. |
| Digital health technologies | Experiences | Experiences patients have had with digital health technologies (apps, wearable devices, websites, medical phone consultations, etc.). Description of tools and what they are used for. Questions participants have about these tools. |
|  | Knowledge | Knowledge about digital health technologies or ideas/ misconceptions of what such technologies might include. |
|  | Positive/motivational factors/enablers | Positive aspects of digital health technologies and motivating factors to use them. |
|  | Negative factors/barriers | Negative aspects of digital health technologies and barriers to using them. |
|  | Data protection | Perception of how secure the data protection of personal health data is in such tools and how important data protection is to the participants. |

**eTable 1: Codebook: Interviews with older adults (continued)**

| **Code** | **Subcode** | **Definition of (Sub)code** |
| --- | --- | --- |
| Digital tools for medication | Experiences | Experiences patients have had with digital tools for medication management (medication intake, medication procurement, providing medication information, etc.). Description of the tools and their purposes. |
|  | Positive | Positive attributes of digital technologies for medication management. |
|  | Negative | Negative attributes of digital technologies for medication management. |
|  | Motivational factors/enablers | All factors that would motivate participants to currently or in the future use digital health technologies for medication management. |
|  | Barriers | All factors currently or potentially preventing participants from using digital health technologies. |
|  | Expectations | Expectations of new digital tools for medication management. Features and functions that would make a tool useful in daily life. |
| (e)*Mediplan^a^* | Current use | Current use and management of a medication plan. |
|  | Knowledge | Knowledge about the *eMediplan* or ideas/misconceptions of the *eMediplan*. |
|  | Positive | Positive features and useful functions of the *eMediplan* or a conventional medication plan. |
|  | Negative | Negative features and useless functions of the *eMediplan* or a conventional medication plan. |
|  | Motivational factors/enablers | Factors that could motivate participants to use an eMediplan. |
|  | Barriers | Factors that could prevent participants from using an *eMediplan*. |
|  | Information | Information that should be included in the *eMediplan* and why. |
|  | Stakeholders | Perception of who should have access to the *eMediplan* and who should keep it up to date. |
|  | Miscellaneous | Additional statements about the *eMediplan* that do not fit into the categories above. |
| Electronic patient record (EPR) | Current use | Current use or non-use of the EPR. |
|  | Knowledge | Knowledge about the EPR or ideas/misconceptions of the EPR |
|  | Positive | Positive features and useful functions of the EPR. |
|  | Negative | Negative features and useless functions of the EPR. |
|  | Motivational factors/enablers | Factors that could motivate participants to use the EPR. |
|  | Barriers | Factors that could prevent participants from using an EPR. |
|  | Miscellaneous | Additional statements about the EPD that do not fit into the above categories. |
| Miscellaneous |  | Interesting text passages that do not belong to any specific code and are likely out of scope. |
| Interesting text passages |  | Interesting text passages that could be quoted because they are well-formulated and/or succinctly express a point. |

^a^ *eMediplan* = Shared electronic medication plan in the Swiss context. Shared electronic medication plans are digital tools designed to manage and share medication plans electronically between healthcare providers and patients.

# **eTable 2: Codebook: Interviews with primary care physicians**

| **Code** | **Subcode** | **Definition of (Sub)code** |
| --- | --- | --- |
| Digital health technologies | Current use | Current use of digital health technologies (e.g., functions in the practice information systems). |
|  | Positive | Positive aspects of currently used digital health technologies, not specifically for medication management. |
|  | Negative | Negative aspects of currently used digital health technologies, not specifically for medication management. |
|  | Expectations | Ideas and expectations about digital health technologies. Suggestions for additional functions and features. |
| Digital tools medication | Current use | Tools for medication management inside and outside of the practice information system that are currently in use. |
|  | Positive | Positive aspects and useful functions of tools for medication management inside and outside of the practice information system. |
|  | Negative | Negative aspects and useless functions of tools for medication management inside and outside of the practice information system. |
|  | Motivational factors/enablers | Factors that motivate the use of digital health technologies for medication management. |
|  | Barriers | Barriers to the use of digital health technologies for medication management. |
|  | Expectations | Expectations for new digital tools for medication management. Features and functions that would make the tool useful in practice. |
| Electronic patient record (EPR) | Current use | Statements on whether primary care physicians are connected to and/or use the EPR. Statements on whether their patients wish to use the EPR. |
|  | Positive | Positive aspects and useful functions of the Electronic Patient Record. Factors facilitating a nationwide implementation. |
|  | Negative | Negative aspects and useless functions of the Electronic Patient Record. Challenges in implementing the EPR nationwide. |
|  | Expectations | Ideas and expectations about the EPR. Suggestions for additional functions and features. |
|  | Miscellaneous | Other statements about the EPR that do not fit into the codes above. |
| (e)*Mediplan^a^* | Management | Current management of the medication plan. How, when, and for what purpose medication plans are prepared. Use of digital tools for creating and managing medication plans. |
|  | Responsibilities | Current task distribution in managing medication plans. Statements about who should have rights and responsibilities when using and managing the *eMediplan* (e.g., primary care physicians, specialists, pharmacists, caregivers, home care services, patients, etc.). |
|  | Information | Information that should or should not be included in the *eMediplan*, and why. |
|  | Positive | Positive aspects and useful functions of the *eMediplan*. Motivating factors for using the eMediplan. |
|  | Negative | Negative aspects and useless functions of the *eMediplan*. Barriers to using the *eMediplan*. |
|  | Expectations | Ideas and expectations about the *eMediplan*. Suggestions for additional functions and features. |

**eTable2: Codebook: Interviews with primary care physicians (continued)**

|  | Nationwide | Responses about whether and how the eMediplan should be implemented nationwide, and whether an obligation would be sensible, including conditions for this. |
| --- | --- | --- |
|  | Miscellaneous | Other statements about the eMediplan that do not fit into the codes above. |
| Medication optimization | Routine | How, when, and for what purpose medication lists are reviewed and optimized and checked with or without patients. Factors that facilitate or hinder regular medication list reviews. |
|  | Documentation | How changes in medications are documented. |
| Digital tools for medication optimization | Current use | Digital tools currently used for reviewing and optimizing medications. |
|  | Positive | Positive aspects and useful functions of digital tools for reviewing and optimizing medications. |
|  | Negative | Negative aspects and useless functions of digital tools for reviewing and optimizing medications. |
|  | Motivational factors/enablers | Factors that might motivate participants to use digital tools for reviewing and optimizing medications. |
|  | Barriers | Factors that might prevent participants from using digital tools for reviewing and optimizing medications. |
|  | Expectations | Ideas and expectations about digital tools for reviewing and optimizing medications. Suggestions for additional functions and features. |
| Medication | Participatory decision making | How patients are currently involved in reviewing and optimizing medications. Advantages and disadvantages of strong patient involvement. Circumstances in which strong or weak patient involvement is possible or sensible. |
| Miscellaneous |  | Interesting text passages that do not belong to any code and are likely out of scope. To be reviewed later. |
| Interesting text passages |  | Interesting text passages that may be quoted because they are well-formulated and/or succinctly express a point. |

^a^ eMediplan = Shared electronic medication plan in the Swiss context. Shared electronic medication plans are digital tools designed to manage and share medication plans electronically between healthcare providers and patients.

# **eFigure 1: Recruitment flowchart: Online survey with older adults**

**322** adults at sampling

**257** adults using ≥2 medications

**252** adults using ≥2 mediations and aged 60-100 years

**65 Excluded**

Did not use ≥2 medications

**5 Excluded**

4 not aged ≥60 years

1 aged >100 years

# **eFigure 2: Recruitment flowchart: Online survey with primary care physicians**

**121** primary care physicians started completing the online questionnaire

**116** fulfilled inclusion criteria

**105** fulfilled inclusion criteria and completed the questionnaire

**5** Excluded

Did not work as a primary care physician

**11** Excluded

Did not completed the questionnaire

**59** Excluded

Randomized into the other part of the questionnaire not relevant for this paper

**46** fulfilled inclusion criteria, completed the relevant part of the questionnaire

# **eTable 3: Baseline characteristics of interviewed older adults**

| Characteristics | n=19 |
| --- | --- |
| Age in years, mean (SD) | 77 (9) |
| Female gender, n (%) | 12 (63) |
| Number of daily prescription medications, mean (SD) | 5 (2) |
| Living situation, n (%) |  |
| Independently at home | 12 (63) |
| Assisted living (services only) | 5 (26) |
| Assisted living (services + care) | 1 (5) |
| Weekly home care | 1 (5) |

# **eTable 4: Baseline characteristics of interviewed primary care physicians**

| Characteristics | n=16 |
| --- | --- |
| Female gender, n (%) | 5 (31) |
| Professional years, mean (SD) | 11 (7) |
| Practice location, n (%) |  |
| City | 13 (81) |
| Suburban or rural area | 3 (19) |
| Individual-/group practice, n (%) |  |
| Group practice | 15 (94) |
| Individual practice | 1 (6) |
| Shared electronic medication plan^a^ integrated in the practice information system, n (%) |  |
| Yes | 9 (56) |
| No | 7 (44) |
| Electronic Patient Record^b^ utilization, n (%) |  |
| Yes | 0 (0) |
| No | 16 (100) |

^a^ Shared electronic medication plans are digital tools designed to manage and share medication plans electronically between healthcare providers and patients (e.g., *eMediplan* in the Swiss context).

^b^ A Swiss Electronic Patient Record is a digital system that securely stores a patients’ health information, enabling authorized healthcare providers to access and update medical data including medication information across practice information systems.

# **eTable 5: Data triangulation**

| **Themes** | **Primary care physicians** | | **Older adults** | |
| --- | --- | --- | --- | --- |
|  | **Survey**  (quantitative findings) | **Interviews**  (qualitative findings, including selected quotes) | **Survey**  (quantitative findings) | **Interviews**  (qualitative findings, including selected quotes) |
| **Current use of medication management IT^a^** | **Different medication management tools are used with widely varying frequency.**  Reported use of:   - shared electronic medication plans^b^: 21 (46%) - Electronic Patient Records^c^: 9 (20%) - other tools to support medication optimization: interaction checker: 33 (72%), apps: 8 (17%), tools that can reconcile various medication lists: 7 (15%), electronic decision support tools: 5 (11%) | **Use of medication management IT is mostly limited to tools integrated into the practice information system.**  Used tools and functions for medication management reported by most participants: creation and editing of medication lists, interaction checker, display of allergies and intolerances, creating and sharing medication plans and prescriptions, connecting to external databases and information sources, storing medication history | **Little use of tools for medication management.**  Reported use of:   - shared electronic medication plan^a^: 7 (3%) - Electronic Patient Record^b^: 12 (5%) | **Little use of tools for medication management.**  Used tools for medication management reported by some participants*:* smartphone applications with pill reminders, medication overviews and/or documentations of medication intake. |
| **Information about existing medication management IT, access and availability** | **Some medication management tools are integrated into the practice information system, while others are currently not available for primary care physicians.**   - Tools available in the practice information system: creation of medication lists: 43 (93%), creation of medication plans: 41 (89%), issuing electronic prescriptions: 32 (70%), other tools: 5 (11%) - Availability of digital tools for medication optimization other than interaction checkers integrated into the practice information system: 18 (39%) - Can not prepare shared electronic medication plans for technical reasons: 7 (29%) | **Some medication management tools are integrated into the practice information system, while others are currently not available for primary care physicians.**   - Tools available in the practice information system: see above - tools missed in the practice information system: pop-up warning for allergies/intolerances/interactions, connection to external databases and information sources, information on delivery shortages, clinical decision support tools, reminder tools, documentation of patients' medication dispensing in the pharmacy, transfer of data from portable devices to the practice information system, display of medications (not) subject to mandatory health insurance coverage. | Limited quantitative data.  Some older adults did not know that Electronic Patient Records existed: 32 (13%)  No data collected about other medication management IT. | **Very few older adults reported to be well informed about existing tools for medication management and optimization.**  Example:  “I had no idea about [the shared electronic medication plan]. […] It was never recommended or offered to me by anyone.” (male, 60) |

**eTable 5: Data Triangulation (continued)**

| **Themes** | **Primary care physicians** | | **Older adults** | |
| --- | --- | --- | --- | --- |
|  | **Survey**  (quantitative findings) | **Interviews**  (qualitative findings, including selected quotes) | **Survey**  (quantitative findings) | **Interviews**  (qualitative findings, including selected quotes) |
| **User-friendliness and support** | No data collected in survey. | **Wish for user-friendly tools and support on how to use such tools.**  Examples:  "[...] it must be easy to handle, so you do not have to learn a lot. Not that you must spend hours figuring it out before everything works. [...]" (male)  "I prefer it when someone just comes and explains it to me, then I can apply it, and that works well." (female) | No data collected in survey. | **Wish for user-friendly tools and support on how to use such tools.**  Examples:  “(Tools) should certainly be programmed in a very, very simple way, especially with getting older and people who are not so well versed.” (female, 64)  “I am glad I am in [a facility for assisted living], where you are much more likely to have help.” (female, 90) |
| **Expected benefit and resulting willingness for future use** | **Varying willingness to use different medication management IT.**   - Willingness to use shared electronic medication plans in the future: 4 (17%) - No willingness to prepare shared electronic medication plans: 3 (13%) - Willingness to use digital tools for medication optimization integrated into the practice information system: 35 (76%) | **Willingness to use medication management IT if it can improve current medication management.**  Examples:  “[…] getting ideas on what else you might need to look at, things that are not on your radar, because everyone has their blind spots, right?" (male)  “But if the digital tool tells me: “The best hypertension treatment according to the guideline is so and so and so”, and I know why I am doing it differently for this patient, that is of absolutely no use to me.” (female) | **Medication management IT is considered useful, and there is a will to use more tools in the future.**   - The Electronic Patient Record improves efficiency in healthcare: 204 (81%) (strongly) agree - The Electronic Patient Record improves quality of patient care: 193 (77%) (strongly) agree - The Electronic Patient Record helps me to better understand my own health: 119 (47%) (strongly) agree - Planning to open an Electronic Patient Record in the future: 68 (49%) - Willingness to use an Electronic Patient Record to manage health information: 164 (68%) (strongly) agree - Willingness to use an Electronic Patient Record to share health information with healthcare providers: 179 (75%) (strongly) agree | **Expected benefit and willingness to use medication management IT in the future when medication becomes complex and more medical and nursing care is required.**  Examples:  “[…] Maybe I will have to take more medication when I get older, then maybe it would help me” (male, 74)  “I think it is important that if you have to go to hospital or if the (home care service comes to your house), that they know what (medication) to give these people so that their medication is not interrupted.” (female, 77)  “In my current [...] health and mental state, I absolutely do not need (such support).” (female, 77) |

**eTable 5: Data triangulation (continued)**

| **Themes** | **Primary care physicians** | | **Older adults** | |
| --- | --- | --- | --- | --- |
|  | **Survey**  (quantitative findings) | **Interviews**  (qualitative findings, including selected quotes) | **Survey**  (quantitative findings) | **Interviews**  (qualitative findings, including selected quotes) |
| **Communication and collaboration in medication management IT** | **Most primary care physicians are open to digital collaboration in medication optimization with other healthcare providers and patients.**  Willingness to use a digital platform to coordinate medication optimization with patients and other healthcare providers: 29 (63%) | **Most primary care physicians are open to digital collaboration in medication optimization with other healthcare providers and patients.**  Example:  “So, [a shared tool] would certainly be cool that physicians, patients, home care, or pharmacies have access to, where everyone can see what [medications] patients are using [and] who has made any changes." (female) | **Most older adults are willing to share their health data digitally with healthcare providers.**  Willingness to use an Electronic Patient Record to share my health information with healthcare providers: 179/240 (71%) (strongly) agree | **Most older adults think that medication management IT can improve communication and therefore their medication management.**  Example:  “Yes, of course it would help me not to forget any [medication]. So, if there are different physicians and different [prescriptions] that might help me if I could look them up somewhere.” (female,83) |
| **Additional workload** | No data collected in survey. | **Primary care physicians fear additional effort without enough benefit**  Example:  “Sometimes it is just annoying because it is badly integrated and […] it eats up your time […].” (male) | No data collected in survey. | **Older adults fear additional effort without enough benefit.**  Example:  “I am too comfortable or too lazy or [my medication] is not important enough to me or I do not get enough benefit from [such tools]” (male, 81). |
| **Data protection** | No data collected in survey. | **Different assessments of data security and its importance.**  Examples:  “Above all, data security. If it is stored in the cloud or simply linked to the patient's surname, first name and date of birth, that is very bad.” (male)  “(It) is always just this data protection story. [...] I think it is generally only positive if you become more open and put data protection a bit in the background.” (female) | **Most older adults are optimistic about data protection.**  The electronic health record stores health data securely: 151 (64%) (strongly) agree | **Different assessments of data security and its importance.**  Examples:  “That is probably one of the most fundamental things, […] that it remains anonymous and that you cannot just go and see what medication [I am] taking.” (male, 74)  “I am not so skeptical about [data protection]. It seems to me, if someone knows what medication I am taking [...], that does not bother me so much.” (female, 90) |

^a^ Medication management IT = Medication management information technologies

^b^ Shared electronic medication plans are digital tools designed to manage and share medication plans electronically between healthcare providers and patients (e.g., *eMediplan* in the Swiss context).

^c^ A Swiss Electronic Patient Record is a digital system that securely stores a patients’ health information, enabling authorized healthcare providers to access and update medical data including medication information across practice information systems.
